# Multimethodological and multiscale investigation of the therapeutic mechanism of Qian Ji Sheng Xue Pian in treating primary immune thrombocytopenia

**DOI:** 10.1186/s41065-025-00620-3

**Published:** 2025-12-06

**Authors:** Yujue Wang, Chengyan Liu, Xiaoqi Sun, Weijie Zhang, Hailin Chen, Wenwei Zhu

**Affiliations:** 1 Department of Hematology, Yueyang Hospital of Integrated Traditional Chinese and Western Medicine, Shanghai University of Traditional Chinese Medicine, 110 Ganhe Road, Shanghai, Hongkou District China; 2 First Department of Oncology, Yueyang Hospital of Integrated Traditional Chinese and Western Medicine, Shanghai University of Traditional Chinese Medicine, Shanghai, China

**Keywords:** Qian Ji Sheng Xue Pian (QJSXP), Primary immune thrombocytopenia, Mendelian randomization, Network pharmacology, Molecular dynamics simulation

## Abstract

**Background:**

For more than 30 years, Qian Ji Sheng Xue Pian (QJSXP) has been used clinically to treat primary immune thrombocytopenia (ITP) with good documented efficacy. However, nothing is known about its underlying mechanisms, effective components, and possible targets. To employ several methodologies to initially investigate the possible targets and therapeutic mechanisms of QJSXP in the treatment of ITP.

**Methods:**

Liquid chromatography-mass spectrometry (LC-MS) identified the principal chemical elements of QJSXP and assessed its probable active components based on ADME characteristics. The research incorporated multidimensional databases to pinpoint probable targets for the active components. Key pathogenic targets linked to ITP were aggregated from several illness databases, and the STRING and Metascape tools were utilized to examine protein interaction activities and related biological processes. Mendelian randomization (MR) was then utilized to determine beneficial targets for the therapy of ITP. The potential targets, including disease targets and MR-positive targets, were found at the intersection, while risk genes were excluded by heterogeneity, pleiotropy, and Steiger analysis to ascertain the core targets. Molecular docking and molecular dynamics simulations were conducted utilizing Schrodinger and Gromacs software to assess the binding affinity of compound-core targets. The toxicological effects of active molecules targeting critical sites were concurrently anticipated using several toxicity databases.

**Results:**

A total of 67 active components and 352 potential targets were discovered in QJSXP, of which 77 were associated with ITP disease targets. Through MR analysis, a total of 12 core genes were identified. Binding scores below − 4.25 kcal/mol constituted 82.0%; docking scores below − 5 kcal/mol represented 60.1%, with an average binding energy of -5.44 kcal/mol. The majority of targets demonstrated strong binding affinity with the components. Toxicity prediction initially highlighted potential hazards, including hepatotoxicity and nephrotoxicity, establishing a foundation for future clinical surveillance.

**Conclusion:**

This study has preliminarily identified the active constituents, associated pathways, and possible targets of QJSXP in the treatment of ITP, offering insights for additional experimental validation of QJSXP’s mechanism of action in ITP.

**Supplementary Information:**

The online version contains supplementary material available at 10.1186/s41065-025-00620-3.

## Introduction

 Isolated peripheral thrombocytopenia (platelet count < 100 × 10⁹/L) without clear, identifiable reasons is the hallmark of primary immune thrombocytopenia (ITP), an acquired autoimmune bleeding condition. About 80% of cases are primary ITP [1, 2]. ITP can present with various clinical symptoms, including severe visceral haemorrhage, mucocutaneous bleeding, life-threatening cerebral haemorrhage, and asymptomatic thrombocytopenia. Additionally, some patients report feeling anxious and exhausted [3, 4]. Loss of immunological tolerance to platelet autoantigens is the main pathophysiology of ITP. This results in aberrant humoral and cellular immunity activation, which together promote rapid platelet destruction and inadequate megakaryocyte platelet synthesis [5]. The incidence rate of ITP in adults ranges from 2 to 10 occurrences per 100,000 individuals annually. Recent research indicates that the incidence rate has risen among young women and senior men [6–8]. The goal of current ITP treatment approaches is to increase platelet production while decreasing platelet destruction. Thrombopoietin receptor agonists (TPO-RAs), rituximab, and corticosteroids are a few examples of clinical therapy. It is possible to use these treatments separately or in combination [9–11]. However, in clinical practice, recurrence and treatment failure are common [9–11].

There are special benefits to using Traditional Chinese Medicine (TCM) to treat ITP. Multi-level targets, a variety of processes, and comparatively low toxicity define complex TCM formulations for ITP, which show great therapeutic benefits and exciting research opportunities. Professors Huang Zhenqiao and Zhou Yongming combined the formulas of Sheng Xue Ling and Shi Hui San to create QJSXP, a hospital-made TCM preparation, based on their years of clinical expertise treating ITP. Sheng Xue Ling, in conjunction with low-dose corticosteroids, has been used extensively for the clinical treatment of ITP for more than thirty years. Sheng Xue Ling’s overall effectiveness rate varied from 85.71% to 95.97% in several clinical trials. At six months, it was far more effective than the prednisone control group (91.07% vs. 53.33%, *P* < 0.01), and there were no notable side effects, which was much less than the hormone group (*P* < 0.05). Platelet counts increased significantly (*P* < 0.05), bleeding symptoms were alleviated or disappeared, bleeding scores decreased, platelet-associated antibodies (PAIgG/A/M) were significantly lower (*P* < 0.05), immune cell subsets (e.g., activated NK cells and T lymphocyte subsets) were normalised, the proportion of platelet-producing megakaryocytes in bone marrow increased (1.75% → 10.91%), and granular megakaryocytes decreased (69.75% → 55.83%) [12–16]. Sheng Xue Ling has been shown in earlier research to control immunological function in ITP patients. In order to exert its immunomodulatory actions, Sheng Xue Ling has been demonstrated in experiments to increase the levels of IL-2 in Th1 cells and IL-10 in Th2 cells [17], modulate NK cells, and improve the function of Ts cells (suppressor T cells) to limit B cell production of PAIg [12]. Additionally, by influencing megakaryocyte progenitor cells, it encourages bone marrow megakaryocytes to differentiate and mature, which increases platelet production and release, decreases capillary fragility, and inhibits bleeding tendencies [12, 18–20]. One technique for halting bleeding is documented in the ancient classic “Wu shi er bing fang (Formularies for 52 Kinds of Disorders)”: “Burn hair and apply it to the affected area.” Charred substances are used as medication in the traditional Shi Hui San formula. Shi Hui San has been shown to improve platelet function in recent experimental trials [21]. Nevertheless, it is still unknown exactly how QJSXP works.

**Fig. 1:**
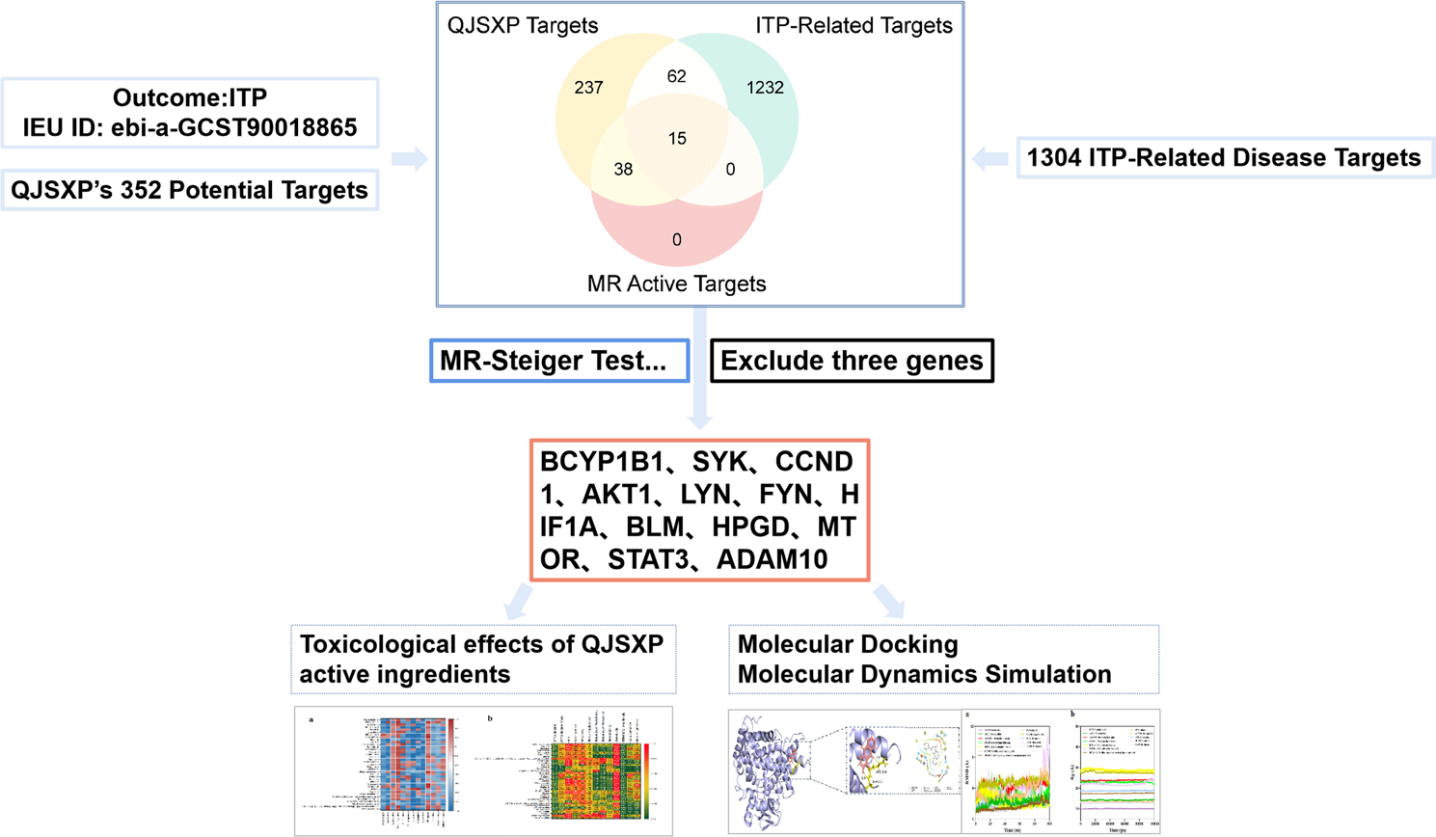
Overview of the study design

The formulae represent a foundational method in TCM therapeutic treatment, guided by the principle of holistic thinking. They employ the synergistic effects of several components, dimensions, and goals, which represent the unique characteristics and benefits of TCM therapeutic practice. Nonetheless, this complexity renders their modes of action challenging to elucidate precisely. Network pharmacology blends transdisciplinary technology and knowledge from systems biology, network biology, and computational biology. It utilizes high-throughput screening, network visualization, and analytical tools to elucidate the intricate biological network connections across medicines, targets, and diseases. This method facilitates a comprehensive and systematic investigation of the interactions between pharmaceuticals and the human organism [22, 23]. MR analysis can forecast drug efficacy and adverse effects using genetic proxies for drug targets, effectively mitigating the influence of reverse causation and confounding variables, thus serving as a robust instrument for drug target validation [24, 25]. Molecular docking is a computational method that predicts binding patterns, binding affinity, and interaction mechanisms between proteins and ligands [26]. When integrated with molecular docking, it helps evaluate the putative mechanisms of pharmacological action. Molecular dynamics simulation assesses the stability, conformational alterations, and interaction energy of receptor-ligand complexes. The integration of both can more systematically evaluate the putative mechanisms of pharmacological action at the computational level [27–29]. This research incorporates mass spectrometry, network pharmacology, and MR analysis, alongside molecular docking and kinetic simulations utilizing Schrödinger and Gromacs software, as well as safety assessments via a multi-toxicology platform (Fig. 1). This study systematically examines the chemical composition, target sites, and signaling pathway mechanisms of QJSXP intervention in ITP at the molecular level, offering comprehensive evidence for its synergistic therapeutic effects through multiple components, targets, and pathways, thereby establishing a foundation for clinical precision medicine and future mechanism-based experimental investigations.

## Materials and methods

### Analysis of the chemical composition of QJSXP

#### Preparation of solutions

Approximately 50 mg of the QJSXP sample was measured. Eight hundred microliters of methanol (Lot No.: 20220115, Manufacturer: Guoyao Vokai Co., Ltd.) were added. The mixture underwent sonication for 15 min, followed by centrifugation at 12,000 rpm for 15 min at 4 °C. The supernatant was transferred to an injection vial for subsequent LC-MS analysis.

#### Conditions for chromatography

The chromatographic column employed was a Waters ACQUITY UPLC·HSS T3 (100 mm × 2.1 mm, 1.8 μm), maintained at a temperature of 40 °C. Elution gradient: flow rate of 0.3 mL/min; injection volume of 7 µL. The mobile phase comprises Phase A (0.1% aqueous formic acid solution) and Phase B (acetonitrile), utilizing a gradient elution protocol. The precise time and ratio configurations are presented in Table 1. The overall flow rate is established at 0.3 mL/min, with an injection volume of 7 µL.

**Table 1:** Gradient elution program for LC-MS analysis

Time	Phase A (0.1% Formic Acid in Water)	Phase B (Acetonitrile)
0	98	2
0.5	98	2
2	95	5
3	85	15
13	50	50
25	5	95
30	5	95
30.5	95	5
35	95	5

#### Conditions for mass spectrometry

Mass spectrometry analysis was conducted using an Orbitrap Elite high-resolution mass spectrometer (Thermo Fisher Scientific). The parameters for the electrospray ionization (ESI) source were established as follows: In positive mode, the ion source temperature is set to 300 °C, with a sheath gas flow rate of 35 arb, an auxiliary gas flow rate of 15 arb, a backflush gas flow rate of 0 arb, a spray voltage of 3.8 kV, a capillary temperature of 350 °C, and an S-Lens RF level of 30%. Negative mode: Ion source temperature 300 °C; sheath gas flow rate 35 arb; auxiliary gas flow rate 15 arb; backflush gas flow rate 1 arb; spray voltage 3.2 kV; capillary temperature 350 °C; S-Lens RF level 60%. The mass detector is a Fourier transform instrument, configured for DDA analysis mode, with an MS1 resolution of 60,000, an MS2 resolution of 15,000, and a mass-to-charge ratio range of 50–1,500.

#### Component evaluation

Obtain the sample solution from section “Preparation of solutions” administer it in accordance with the chromatographic and mass spectrometric parameters outlined in sections “Conditions for chromatography” and “Conditions for mass spectrometry” utilize Compound Discoverer 3.3 software, implement the natural products template, correlate the first- and second-level mass spectra from the sample with the compounds in the Otcml database, and ascertain the unidentified compounds in QJSXP.

### Network pharmacology mechanism prediction of QJSXP in ITP treatment

#### Identification of potential active components and targets of QJSXP

The structural formulas were obtained from the PubChem database (https://pubchem.ncbi.nlm.nih.gov) based on the chemical components identified through mass spectrometry analysis [30]. The structures were subsequently entered into SwissADME (http://www.swissadme.ch) for initial screening of potential active components [31]. The criteria for screening were established as follows:1. The predicted result for gastrointestinal absorption is “High,” indicating that the ingredient has good oral bioavailability; 2. At least two of the five pharmacokinetic rules (Lipinski, Ghose, Veber, Egan, Muegge) predict a result of “Yes”; 3. Bioavailability Score ≥ 0.55 [32]. Potential targets for the components that passed the bioavailability screening were predicted using SwissTargetPrediction (http://swisstargetprediction.ch) [33]. Targets with a probability exceeding 0.5 were identified as initial potential targets. Additionally, target prediction was enhanced through the use of PharmMapper and SuperPred databases [34, 35]. The predicted targets from various sources were deduplicated and consolidated to form the potential targets of the active components of QJSXP. Further target screening was conducted for other identified compounds. In parallel, established targets of potential active compounds that were not identified through computational methods were added based on the current published literature.

#### Compilation of ITP disease targets

Targets associated with ITP disease were obtained by querying the GeneCards database (https://www.genecards.org), OMIM database (https://www.omim.org), DRUGBANK database (https://go.drugbank.com), GAD database (https://maayanlab.cloud/Harmonizome/resource/Genetic+Association+Database), DisGeNET database (https://disgenet.com), and HERB database (http://herb.ac.cn), utilising “primary immune thrombocytopenia” as the search term [36–41]. Upon consolidating the targets derived from these six illness databases, redundant items were eliminated to produce the definitive set of ITP disease targets. The selected disease targets were subsequently standardised and unified via the UniProt database (https://www.uniprot.org) for consistent naming [42].

#### Development of protein-protein interaction network and pathway enrichment analysis

To clarify the relationships between the potential targets of QJSXP’s active components and the targets associated with ITP disease, the overlap of these two sets of targets was identified. The identified common targets were submitted to the STRING database (https://cn.string-db.org) to develop a protein-protein interaction (PPI) network model [43]. The species was designated as “Homo sapiens”, with all other parameters remaining at their usual defaults. This procedure produced the PPI network. Gene enrichment analysis of the shared targets was conducted utilising the Metascape platform (https://metascape.org/gp/index.html) [44]. This study encompassed Gene Ontology (GO) terminology, including Biological Process (BP), Cellular Component (CC), and Molecular Function (MF), in addition to Kyoto Encyclopaedia of Genes and Genomes (KEGG) pathways.

#### Development of the QJSXP component-ITP target-pathway network

The component-ITP target-pathway network of QJSXP was developed utilising Cytoscape 3.10.0 [45]. The Network Analyser plugin in Cytoscape was utilised to assess the network topological metrics of the active components and targets, including degree, betweenness centrality, and closeness centrality. These characteristics were employed to identify significant prospective targets and primary active components that may contribute to the therapeutic outcomes.

### Mendelian randomisation analysis to investigate the potential causal relationship between potential QJSXP targets and ITP outcomes

Cis-eQTL data for 216 prospective targets, predominantly sourced from peripheral blood samples, were acquired from eQTLgen (https://eqtlgen.org) [46]. Clumping was executed with settings configured to R² = 0.3 and kb = 100. ITP (IEU ID: ebi-a-GCST90018865) served as the outcome, with summary-level data acquired from the GWAS database (https://www.ebi.ac.uk/gwas) [47]. The effect alleles of exposure and result SNPs were standardised to guarantee uniform effect directions, and SNPs with discordant alleles were eliminated. The variance in the exposure attributed to the SNPs was assessed using the F-statistic, and instruments with an F-statistic less than 10 were omitted to prevent weak instrument bias. Additionally, Steiger directionality testing (Steiger filtering) was utilised for the SNPs. Only instruments demonstrating a superior R² for the exposure (R²_GX) compared to the outcome (R²_GY) and a Steiger test p-value significantly endorsing the proposed causation direction (*P* < 0.05) were preserved. This filtering stage aids in eliminating instrumental variables that could contravene the fundamental assumptions of Mendelian Randomisation due to reverse causation or predominant influence on the outcome, hence bolstering the validity of the deduced causal direction. The Inverse Variance Weighted (IVW) method served as the principal analytical approach for MR. Supplementary studies were performed utilising MR-Egger regression, Weighted Median, and Weighted Mode methodologies to investigate horizontal pleiotropy, heterogeneity, and assess robustness against certain invalid instruments. The False Discovery Rate (FDR) correction was implemented to address repeated testing, with results with a q-value < 0.05 being statistically significant. This extensive MR methodology sought to discover possible treatment targets of QJSXP with probable causal links to ITP outcomes.

### Molecular docking analysis

Core therapeutic targets were determined by intersecting results from network pharmacology and Mendelian randomisation analyses. UniProt entry IDs were obtained for each target and utilized to acquire high-resolution three-dimensional structures from the RCSB PDB (https://www.rcsb.org) [42]. Priority was assigned to X-ray structures containing co-crystallized ligands to facilitate benchmarking and hotspot identification. The production of ligands entailed downloading 37 active phytochemical structures from PubChem and turning them into 3D conformers. The ligands were subsequently prepared using Schrödinger (LigPrep) [48]. Protonation states at physiological pH were designated, tautomers and stereoisomers were examined, explicit hydrogens were incorporated, and geometries were subjected to energy minimization. Partial charges suitable for later molecular dynamics parameterization(CGenFF) were acquired. To facilitate direct comparison, established inhibitors utilized as docking controls (e.g., alisertib) were produced using the identical ligand preparation method (protonation state assignment, conformer optimization, and parameterization) as the phytochemicals. Protein preparation involved the processing of each PDB structure using the Schrödinger Protein Preparation Wizard, where feasible, missing side chains and loops were modeled, bond orders were established, protonation states were assigned, and crystallographic waters beyond 5 Å from the binding site were eliminated, while functionally significant structural waters were preserved. Co-crystallized ligands were retained for benchmarking runs to facilitate hotspot residue mapping. The docking technique and pose selection were executed via Schrödinger Glide in SP mode. Grids were centered on the centroid of the co-crystallized ligand when applicable, with a grid box sufficiently expansive to encompass essential nearby subsites. Control ligands were docked using the same parameters (software, receptor conformation, and grid box) as the phytochemicals. We maintained the three lowest-energy conformations for each receptor-ligand pair for examination. The criteria for pose selection integrated docking scores with interaction feasibility, favoring poses that replicate experimentally identified pharmacophores, interact with hotspot residues, and exhibit geometries aligned with established inhibitors, rather than depending solely on scores. All chosen docking postures were exported as PDB files and examined in PyMOL (version 3.1) to see binding interactions and confirm hotspot engagement. Benchmarking and validation involved redocking and enrichment analysis. During redocking, each co-crystallized ligand was extracted and prepared in the same manner as the phytochemicals, then redocked into the respective receptor grid. The ligand heavy-atom root mean square deviation (RMSD) was determined after aligning the protein backbone, with an RMSD of less than 2.0 Å considered indicative of a successful reproduction of the experimental pose. In enrichment analysis, curated sets of actives and decoys were docked for specific targets, and ROC AUC along with early enrichment factors (EF1–5%) were computed to evaluate scoring discrimination, with AUC >0.85 and EF >10 serving as acceptance criteria for advancing to comprehensive phytochemical screening.

### Molecular dynamics simulations

Chosen receptor–ligand complexes (ranked by combined docking score and hotspot interaction) and their respective control complexes underwent molecular dynamics simulations utilizing Gromacs 2022. The CHARMM36 force field was employed for proteins, whereas ligand parameters were generated using the CGenFF workflow [49]. TIP3P explicit water was employed for solvation. The system setup entailed positioning each complex at the center of a cubic box with a minimum padding of 1.0 nm, solvation with TIP3P water, neutralization using Na⁺/Cl⁻ ions to achieve an ionic strength of around 0.15 M, and the implementation of periodic boundary conditions in all directions [50].

Minimization and equilibration were conducted utilizing steepest-descent minimization (3,000 steps) succeeded by conjugate-gradient minimization (2,000 steps) within a staged restraint protocol that included (i) solvent minimized with protein/ligand restrained, (ii) ions relaxed with restraints, and (iii) the complete system minimized without restraints. Equilibration using NVT (temperature coupling at 310 K) followed by NPT (pressure coupling at 1 bar) ensembles, with positional restrictions progressively alleviated. Production runs were conducted as 100 ns molecular dynamics simulations with a 2 fs timestep utilizing the leapfrog integrator. All covalent bonds involving hydrogen atoms were constrained using the SHAKE algorithm. Long-range electrostatics employed PME with a 1.0 nm real-space cutoff. Trajectory analysis was conducted following the exclusion of initial non-equilibrated frames, utilizing Gromacs tools to calculate RMSD (for protein backbone and ligand heavy atoms), per-residue root mean square fuctuation (RMSF), radius of gyration (Rg), solvent accessible surface area (SASA), hydrogen bond counts and occupancies, as well as MM-PBSA binding free-energy estimates, with frames sampled every 100 ps from the concluding 20 ns. To evaluate the stability of ligand binding and the preservation of essential interactions, time-resolved hydrogen-bond profiles were created for each phytochemical–protein complex and its respective control complex. Hydrogen bonds are characterized by a donor–acceptor distance of ≤ 3.5 Å and an angle of ≥ 120°. In this work, alisertib functioned as the control compound in molecular dynamics simulations. These controls were produced and docked under conditions identical to those employed for phytochemicals, offering comparison reference points for binding pose geometry and interaction patterns. Comparative metrics for each phytochemical–protein pair were derived by conducting identical analyses on the corresponding control complex. Subsequently, final values (averages ± SD) for backbone RMSD, pocket RMSF, Rg, H-bonds, and ΔG_bind were compared, and statistical tests were applied as necessary. Reported data represent means ± SD throughout the final 20 ns, with equilibration assessed from the RMSD traces. Controls were incorporated for each target using an accessible co-crystallized ligand or recognized therapeutic inhibitor, with the relevant ligand (e.g., alisertib) functioning as the control in the MD processes. These controls were produced and docked under conditions same to those employed for phytochemicals, offering comparison reference points for posture geometry and interaction patterns.

### Toxicological prediction

Screen the core components that act on the core target points from the components obtained by mass spectrometry. Upload the 2D structures of the components to ProTox (https://tox.charite.de/protox3/), pkCSM Toxicity (https://biosig.lab.uq.edu.au/pkcsm/), and ADMETlab3.0 (https://admetlab3.scbdd.com/) to predict the acute oral toxicity(LD50), organ-specific toxicity(hepatotoxicity, nephrotoxicity, neurotoxicity, cardiotoxicity, etc.), mutagenicity, immunotoxicity, and other properties. The use of multiple toxicity prediction tools reduces the number of false positives or false negatives, which leads to more dependable toxicity results [51–53].

## Results

### Screening of active components and identification of potential targets of QJSXP by mass spectrometry

LC-MS analysis initially produced 183 results in negative ion mode and 275 in positive ion mode. Following first filtration according to oral bioavailability (OB) and drug-likeness (DL) criteria, 266 chemical constituents were discovered. Subsequent target prediction and additional active component screening finally found 352 potential targets and 67 potential active components. Detailed results of mass spectrometry analysis are provided in the supplementary materials (Figure S1,S2, Table S1).

### Compilation of ITP disease targets

Targets for ITP illness were obtained from six databases: 679 from GeneCards, 597 from OMIM, 181 from DrugBank, 23 from GAD, 36 from DisGeNET, and 92 from HERB. The amalgamation of targets from these six datasets yielded an initial pool of 1608 targets. Following deduplication, 1304 distinct ITP-related targets were acquired. The intersection of the 352 potential targets of QJSXP’s components (from Section "Screening of active components and identification of potential targets of QJSXP by mass spectrometry") and the 1304 ITP illness targets yielded a final set of 77 shared targets.

### Construction and enrichment analysis of the PPI network

**Fig. 2:**
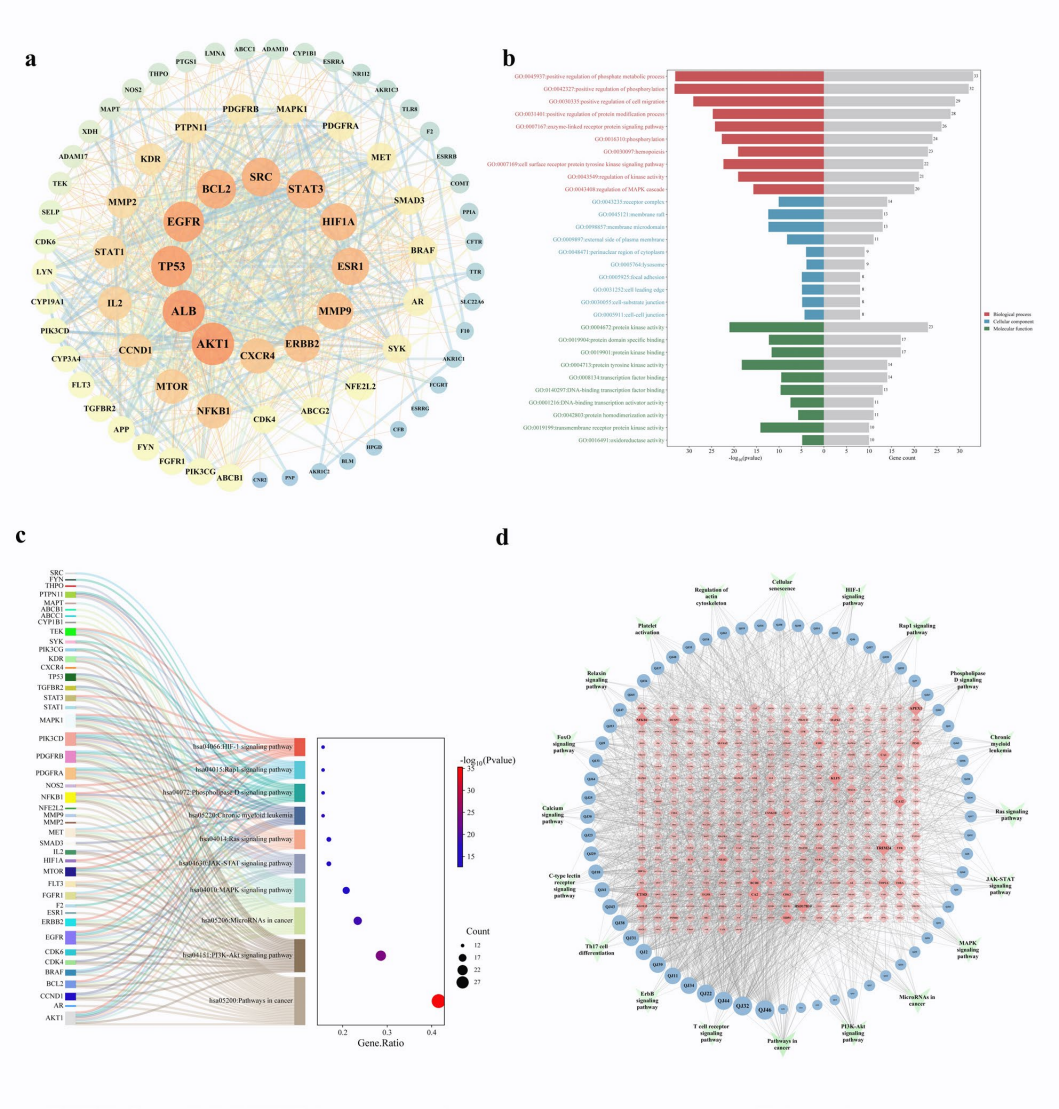
**a** PPI network after Cytoscape processing; (**b**) GO enrichment; (**c**) KEGG pathway enrichment; (**d**) QJSXP Active Component-ITP Common Target-Pathway Network(○ represents active ingredients, ♢ represents common targets, ➤ represents Pathway), Edges connecting nodes signify interactions. In the network visualisation, increased node size and a deeper hue signified a heightened potential influence on ITP

Cytoscape was used to create a PPI network for the 77 common targets (Fig. 2a). The leading 20 putative targets, ranked by Degree value inside the network, including essential proteins including AKT1, ALB, TP53, EGFR, BCL2, and STAT3, among others. Results from the Metascape gene enrichment analysis demonstrate that these targets are significantly linked to various biological processes, such as the positive regulation of phosphate metabolism, cell migration, protein modification, and the enzyme-linked receptor protein signaling pathway (Fig. 2b). Significantly enriched key signalling pathways included Pathways in Cancer, the PI3K-Akt signalling pathway, MicroRNAs in Cancer, the MAPK signalling pathway, the JAK-STAT signalling system, and the Ras signalling pathway, among others (Fig. 2c). The targets associated with modifying ITP were predominantly concentrated in molecular functions including protein kinase activity, specific protein domain binding, protein kinase binding, and transcription factor binding(Comprehensive target and KEGG pathway enrichment data are provided in Supplementary table S2 and Figure S3).

### Development of the QJSXP active component-ITP common target-pathway network

The developed network comprised 433 nodes and 2187 edges (Fig. 2d).The network analysis results indicated that each active component engaged with numerous potential targets and participated in various signalling pathways, whereas each target was linked to multiple components. Luteolin had a high degree (84), betweenness centrality (0.0773), and closeness centrality (0.4095) among the nodes, signifying its potential as a principal active component. Additional essential components comprised Ellagic acid, Kaempferol, Apigenin, and Eupafolin, among others (Table S3).

### Pharmacological target MR analysis and identification of principal targets

**Fig. 3:**
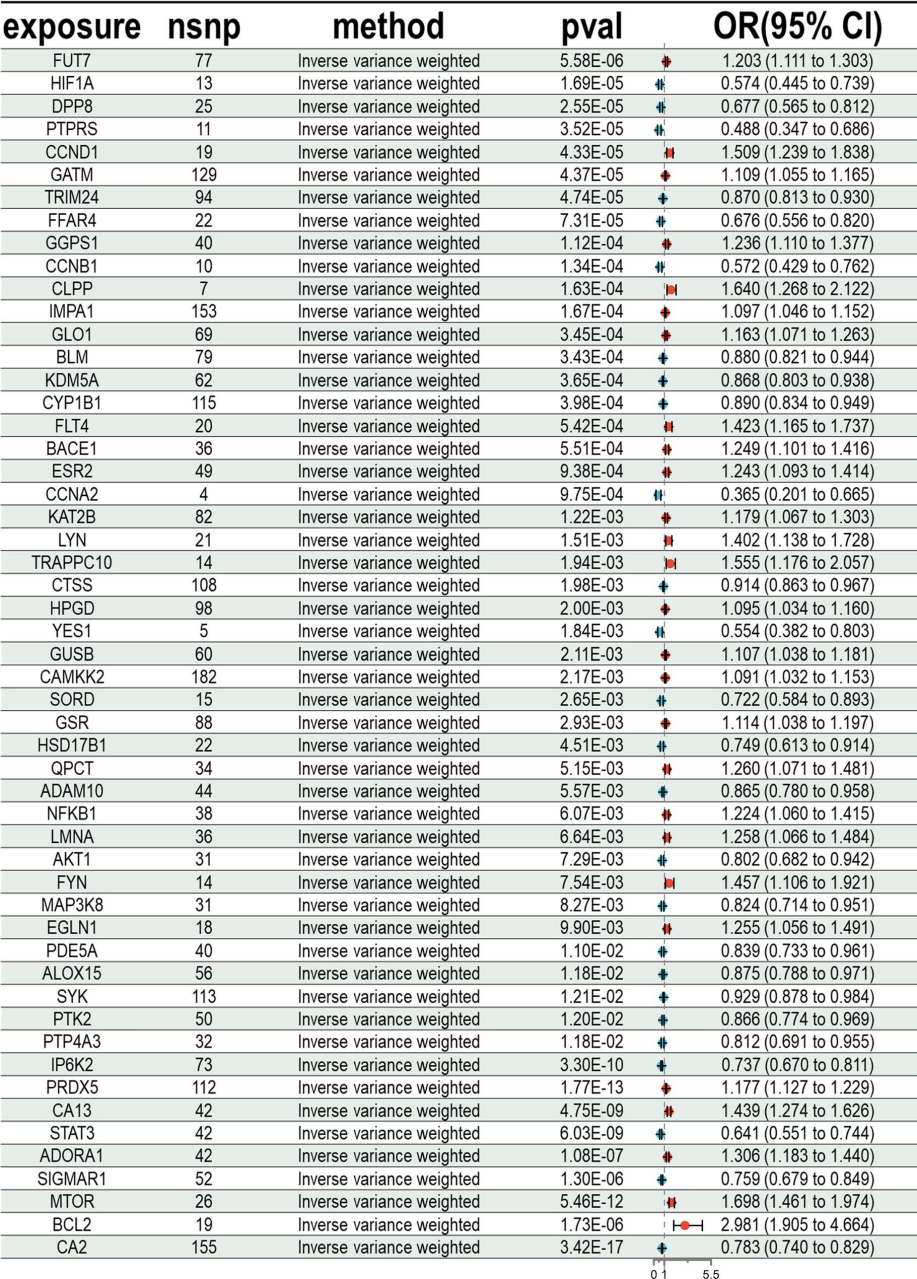
The forest plot illustrates the outcomes of 52 putative druggable genes

**Fig. 4:**
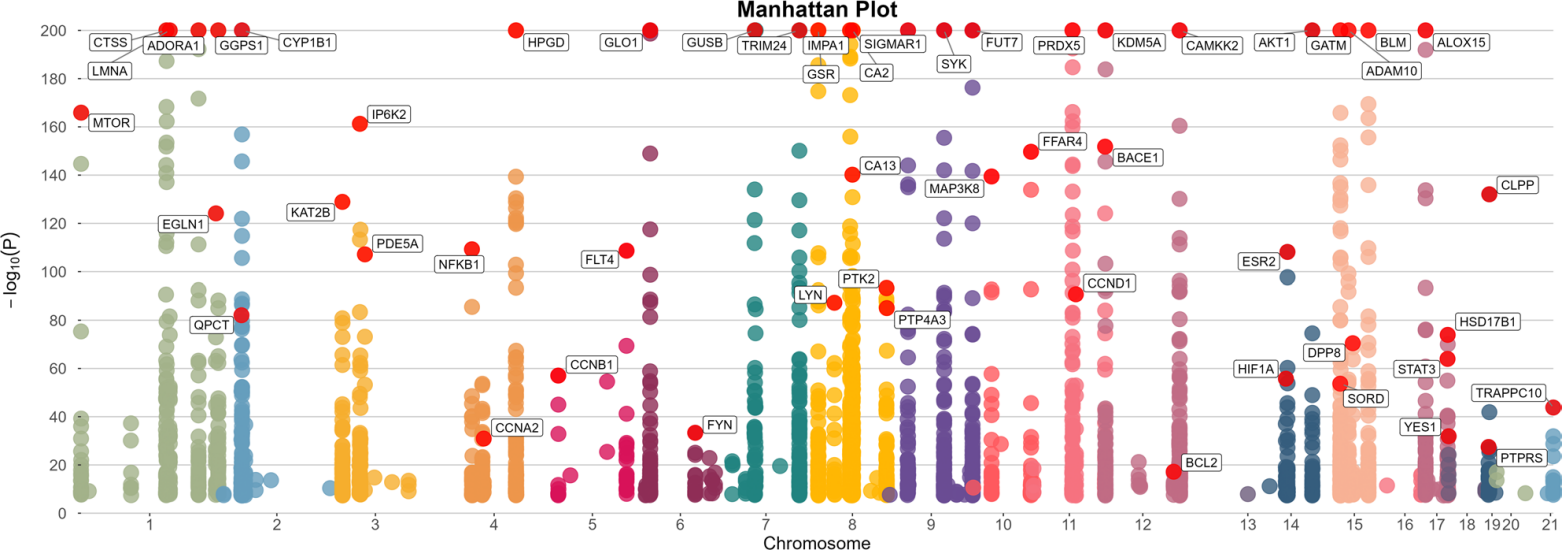
Manhattan plot depicting outcomes from the drug-target Mendelian randomisation analysis for ITP

In the primary MR analysis, each gene, indicated by its cis-eQTL as a surrogate for alterations in gene expression levels, was examined for its causal association with ITP risk. Following FDR correction, 53 genes (FDR < 0.05) were identified as potentially causally linked to ITP (Figs. 3 and 4). Of these, 25 genes were linked to an elevated risk of ITP (OR > 1), whereas 28 genes were linked to a reduced risk of ITP (OR < 1).The intersection of the 77 common targets identified in network pharmacology (Section "Compilation of ITP disease targets") with 53 MR-positive genes resulted in 15 core genes. After conducting heterogeneity and pleiotropy tests, as well as Steiger analysis, three risk genes were excluded, resulting in 12 core targets (CYP1B1, SYK, CCND1, AKT1, LYN, FYN, HIF1A, BLM, HPGD, MTOR, STAT3, ADAM10) linked to 37 active components. The MR analysis results are robust, indicating a potential causal link between the QJSXP target and ITP.

### Results of molecular docking

#### Docking protocol validation and control confirmation

A benchmarking analysis was conducted to validate the reliability of the docking method before docking the phytochemicals. Co-crystallized ligands for each protein target were redocked into their corresponding active sites utilizing the identical docking procedure. The RMSD values between redocked and crystallographic positions were consistently below 2 Å, validating that the docking method correctly replicated experimentally reported binding modes (Figure S4a). The docking confirmation for the control inhibitor alisertib demonstrated occupancy of the anticipated pocket, with a docking score of − 8.75 kcal/mol, and redocking yielding heavy-atom RMSDs of 1.6 Å and 1.8 Å, both below 2.0 Å (Figure S4b, Figure S4c).

Furthermore, enrichment analysis was conducted by docking a dataset of known active and decoy chemicals with selected targets. The findings exhibited substantial predictive capability, with area under the receiver operating characteristic curve values surpassing 0.85 and enrichment factors (EF) reaching 10, signifying robust differentiation between active and inactive compounds. The dual validation (redocking and enrichment analysis) affirmed the integrity of the docking grid parameters and the dependability of the ensuing phytochemical docking results. The control docking results further validate the appropriateness of the chosen grid and parameter configurations for the phytochemical docking initiative.

#### Molecular docking outcomes and residue-level analysis

**Fig. 5:**
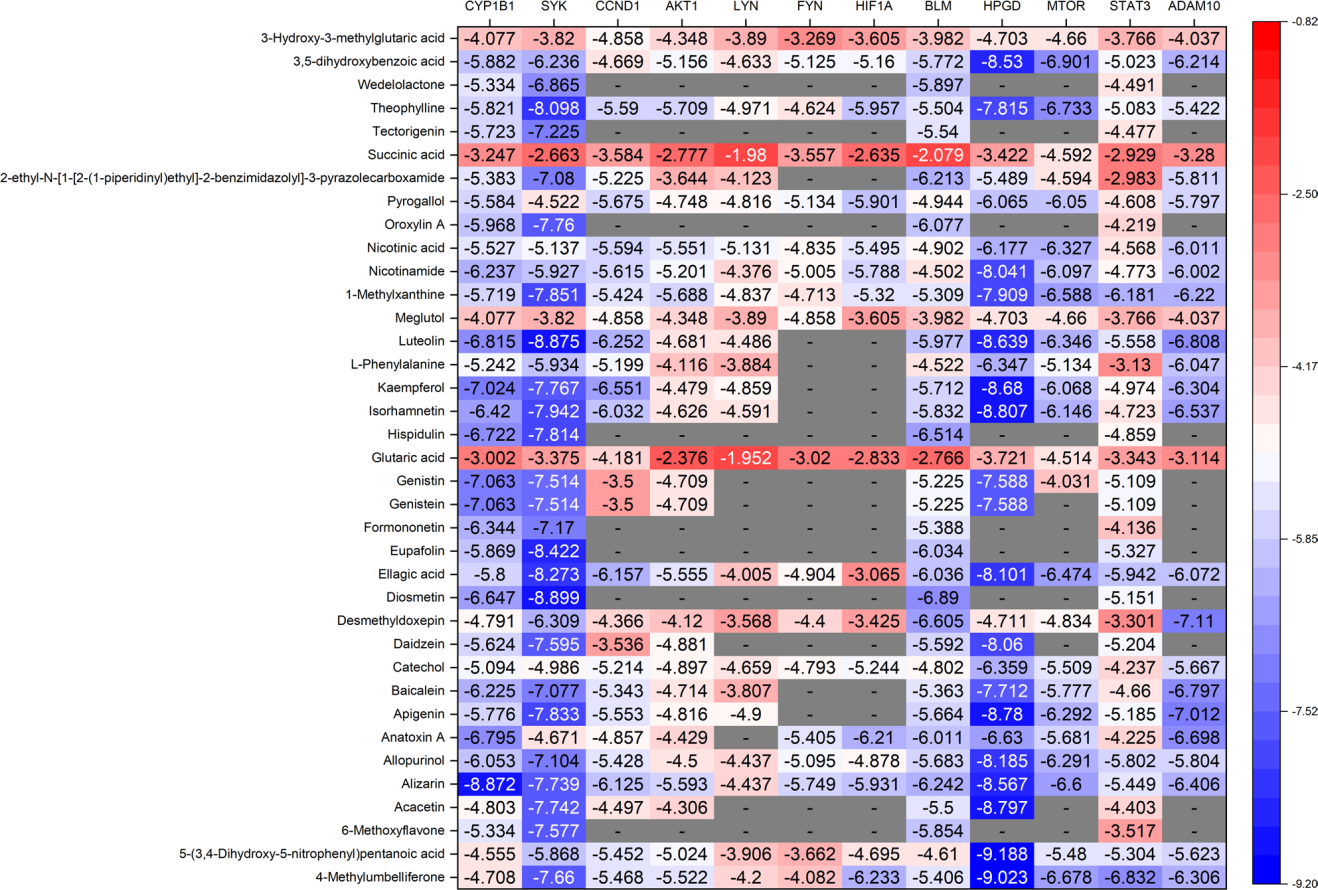
Heatmap of pairwise molecular docking outcomes between core risk gene products and the principal active components of QJSXP. Decreased binding energy values suggest enhanced predicted interactions between the compound and the gene product

**Fig. 6:**
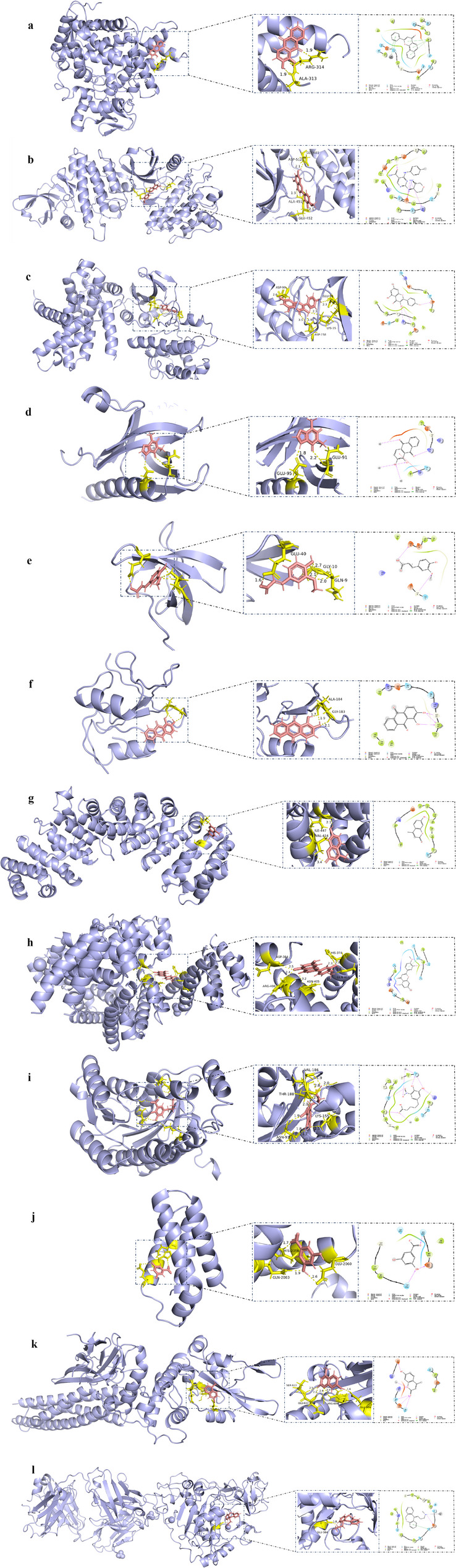
Molecular docking of the principal active components of QJSXP with core risk genes. **a** Alizarin docking CYP1B1; (**b**) Diosmetin docking SYK; (**c**) Kaempferol docking CCND1; (**d**) Theophylline docking AKT1; (**e**) Nicotinic acid docking LYN; (**f**) Alizarin docking FYN; (**g**) 4-Methylumbelliferone docking HIF1A; (**h**) Diosmetin docking BLM; (**i**) 5-(3,4-Dihydroxy-5-nitrophenyl)pentanoic acid docking HPGD; (**j**) 3,5-dihydroxybenzoic acid docking MTOR; (**k**) 4-Methylumbelliferone docking STAT3; (**l**) Desmethyldoxepin docking ADAM10

From the docking of 12 core targets with 37 active components, 444 receptor-ligand pairings were generated, yielding 346 legitimate docking results (Fig. 5). Among these, 284 pairs (82.0%) exhibited docking scores below − 4.25 kcal/mol; 208 pairs (60.1%) demonstrated scores below − 5 kcal/mol; and 47 pairs (13.6%) recorded scores below − 7 kcal/mol. The molecular docking data demonstrate that a higher negative score correlates with increased affinity and enhanced interaction between the receptor and ligand. The highest binding score was recorded for HPGD with 5-(3,4-Dihydroxy-5-nitrophenyl)pentanoic acid (−9.188 kcal/mol), followed by 4-Methylumbelliferone (−9.023 kcal/mol), and for SYK with Diosmetin (−8.899 kcal/mol). The target proteins CYP1B1, SYK, BLM, and STAT3 exhibited binding affinities with various constituents of QJSXP, yielding an average binding score of −5.12 kcal/mol. Alizarin and 1-Methylxanthine demonstrated significant binding affinity with various targets among the ligands. Residue-level analysis indicated that diosmetin interacted with SYK via Asp512, Ala451, and Glu452, while kaempferol was associated with CCND1 at Lys35 and Glu94. These residue-specific interactions offer mechanistically validated targets for the anticipated binding mechanisms of phytochemicals. Fig. 6 illustrates the molecular docking results between the core risk gene products and the principal active components. Further docking information is included in Supplementary figure S5.

### Outcomes of molecular dynamics simulation

**Fig. 7:**
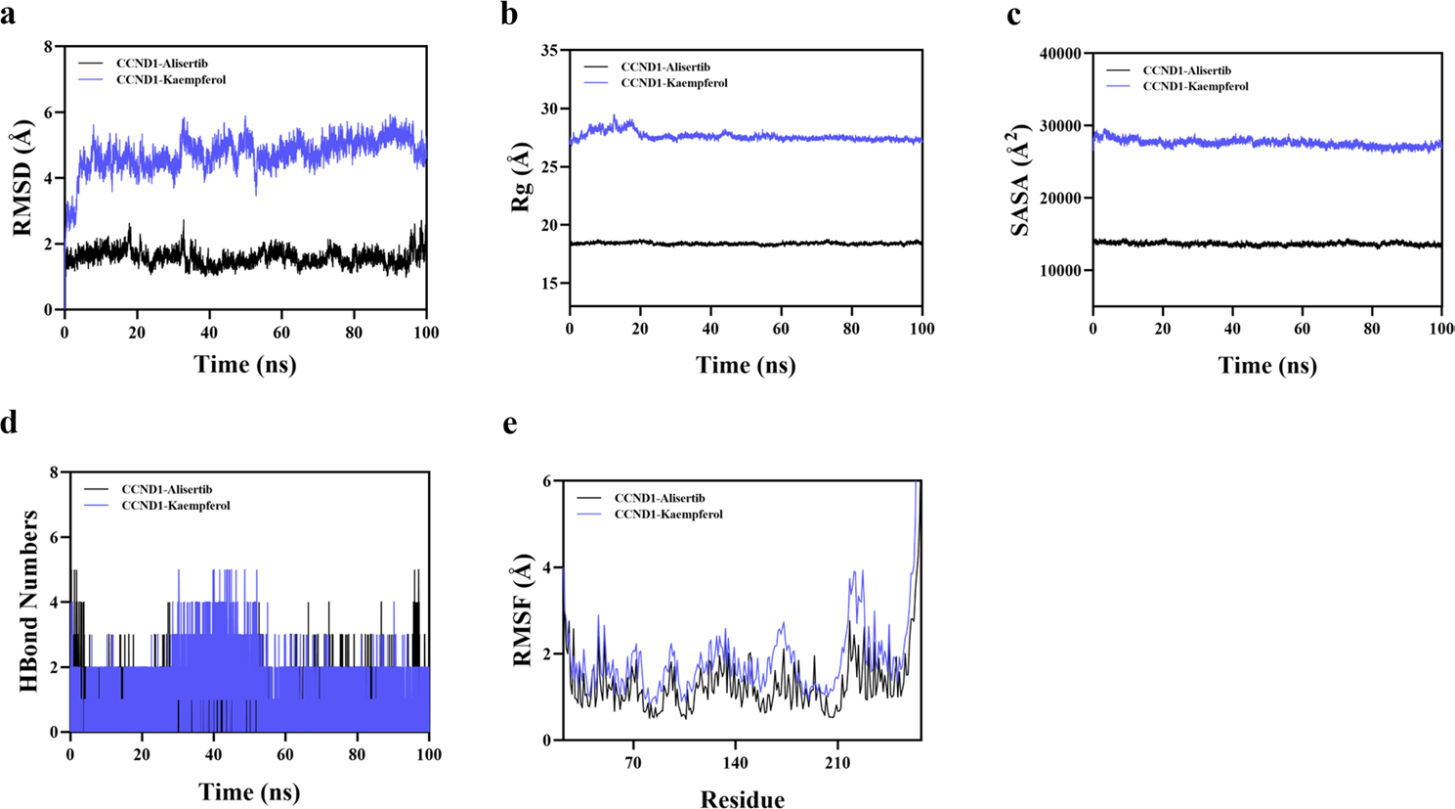
Molecular dynamics simulation results of CCND1 complexes with the inhibitor alisertib (black) and the phytochemical kaempferol (blue) over 100 ns. **a** The RMSD value of the protein-ligand complex as a function of time. **b** Temporal variation of the Rg for the protein-ligand combination. **c** Temporal analysis of the SASA of the protein-ligand complex. **d** Temporal variation in the quantity of hydrogen bonds within the protein-ligand complex. **e** RMSF of the protein-ligand combination

**Fig. 8:**
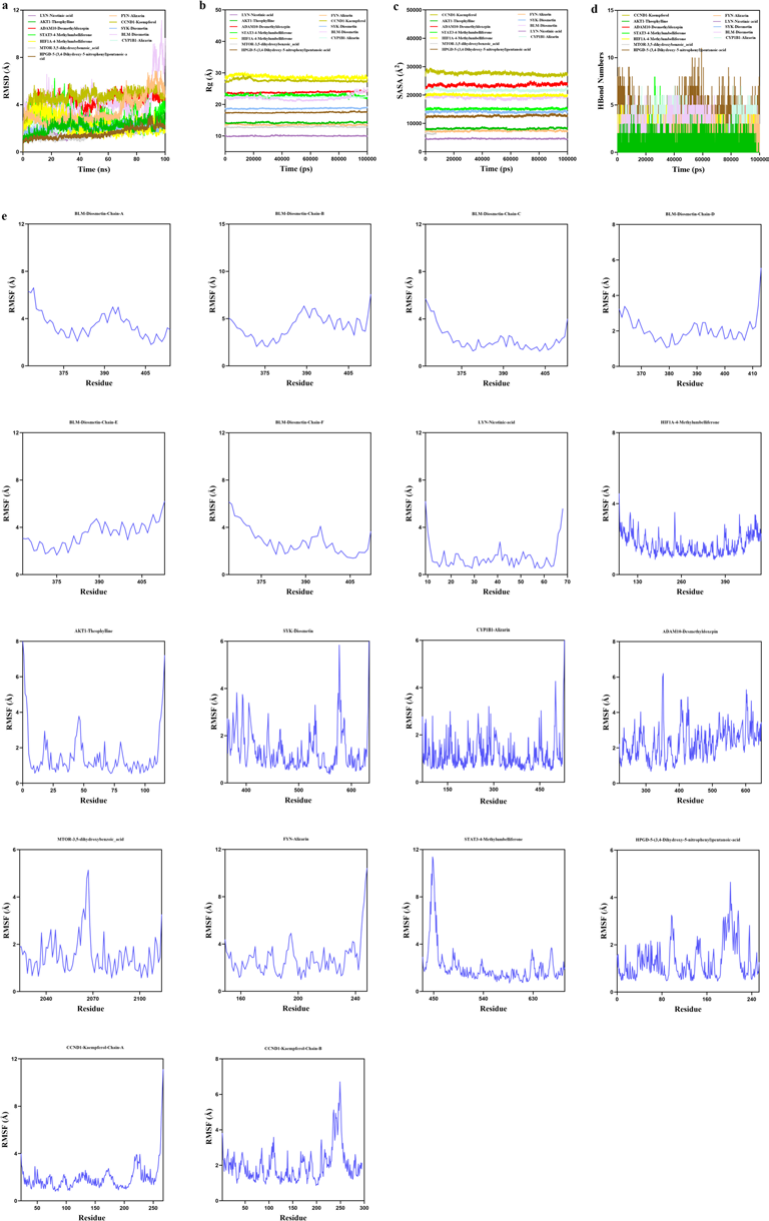
Analysis of Molecular Dynamics Simulations of the Protein-Ligand Complex in QJSXP. **a** The RMSD value of the protein-ligand complex as a function of time. **b** Temporal variation of the Rg for the protein-ligand combination. **c** Temporal analysis of the SASA of the protein-ligand complex. **d** Temporal variation in the quantity of hydrogen bonds within the protein-ligand complex. **e** RMSF of the protein-ligand combination

RMSD is a dependable metric for evaluating the conformational stability of proteins and ligands, reflecting the variation of atomic locations from their original coordinates. A reduced divergence indicates enhanced structural stability. To specifically evaluate whether the ligand remains stably bound in the pocket and retains tight interactions, the phytochemical kaempferol bound to CCND1 was directly compared with the reference inhibitor alisertib under identical MD conditions. When alisertib is used as a control, the QJSXP phytochemical complex has binding stability and residue-level interaction patterns similar to those of a validated inhibitor, but it exhibits a marginally weaker MM-PBSA-derived ΔG_bind. The comparative MD metrics (RMSD, RMSF, Rg, ΔG_bind) enhance the structure-based evaluation and offer direction for prioritizing drugs for experimental validation (Figure S4). The CCND1–alisertib complex rapidly converged to a backbone RMSD of ~ 1.5–2.0 Å and remained stable, whereas the CCND1–kaempferol complex equilibrated at a higher RMSD (~ 4–5 Å) without progressive drift (Fig. 7a). For CCND1, the Rg and SASA profiles of both complexes were essentially constant, indicating no large-scale unfolding or collapse of the protein scaffold (Figs. 7b and c). Hydrogen-bond monitoring further revealed that kaempferol maintained approximately 2–4 ligand–protein hydrogen bonds for most of the simulation, while alisertib exhibited a more intermittent pattern (0–3 hydrogen bonds) (Fig. 7d). The RMSF profiles of the two complexes were highly similar, with only slightly increased fluctuations in loop and terminal regions in the kaempferol-bound system (Fig. 7e). These observations demonstrate that kaempferol remains stably accommodated within the CCND1 binding pocket, supported by a persistent hydrogen-bond network comparable to that of the inhibitor. Fig. 8a indicates that the HPGD-5-(3,4-dihydroxy-5-nitrophenyl)pentanoic acid complex system attained equilibrium after 5 ns and then exhibited fluctuations around 2.3 Å. Small molecule constituents, including theophylline, desmethyldoxepin, and 4-methylumbelliferone, exhibited considerable stability when associated with target proteins such as CCND1, AKT1, and ADAM10. Subsequent analysis indicated that the Rg and SASA of complex systems such as CCND1-Kaempferol and AKT1-Theophylline exhibited minor variations throughout the simulation (Figs. 8b and c), suggesting that conformational changes transpired during the dynamic process. Hydrogen bonds are essential in ligand-protein interactions, and all twelve protein-ligand complexes exhibited sustained hydrogen-bonding interactions during the 100 ns simulations. Fig. 8d depicts the number of hydrogen bonds established between the small molecules and target proteins during the dynamic process. Most complexes maintained approximately 2–4 hydrogen bonds over time, while the HPGD–5-(3,4-dihydroxy-5-nitrophenyl)pentanoic acid complex often displayed over 5 concurrent hydrogen bonds, signifying a robust interaction network and stable positioning of the ligand within its binding pocket. RMSF signifies the flexibility of amino acid residues in proteins. Fig. 8e demonstrates that the RMSF values for complex systems, such as SYK-Diosmetin, AKT1-Theophylline, and ADAM10-Desmethyldoxepin, were largely low (mainly below 4 Å), signifying diminished flexibility and increased stability. In summary, the twelve complex systems demonstrated stable binding, robust hydrogen bonding interactions, and generally favourable binding between the active components and their target proteins.

### Toxicology forecast of QJSXP

**Fig. 9:**
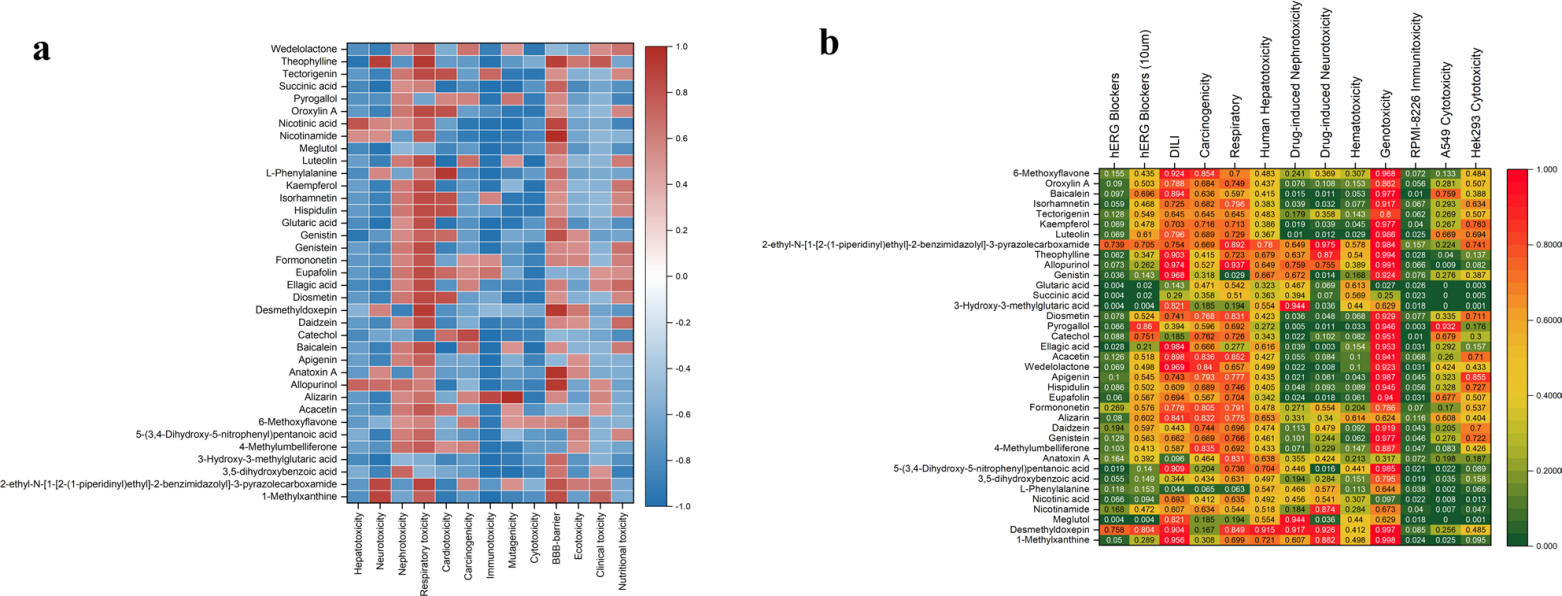
Heatmap of the potential toxicity forecast for QJSXP. **a** ProTox predicted toxicity is displayed by color: red for toxic (active) and blue for non-toxic (inactive). Color intensity represents the strength of the prediction. **b** The predicted toxicity from ADMETlab 3.0 is illustrated by a gradient of green, yellow, and red: green signifies negligible or low risk, yellow denotes moderate danger, and red suggests a potential high risk. The shift from green to red signifies an escalating likelihood of hazardous risk

ProTox projections (Fig. 9a) suggested that QJSXP’s toxicity was mostly linked to nephrotoxicity, respiratory toxicity, and potential effects on the blood-brain barrier (BBB). Among these, pulmonary damage was anticipated to be especially pronounced. Moreover, substances such as 3,5-dihydroxybenzoic acid, baicalein, luteolin, and apigenin were anticipated to demonstrate toxicity in a higher number of experiments. The ADMETlab3.0 predictions (Fig. 9b) indicate that the primary toxicities of the components were focused on genotoxicity, drug-induced liver injury (DILI), carcinogenicity, and respiratory toxicity. In contrast, the anticipated cytotoxicity for A549 and HEK293 cell lines was predominantly low, with A549 displaying considerable variability among compounds, while HEK293 consistently demonstrated a steady low-risk profile. Table 2 demonstrates that the majority of components displayed low bioconcentration factor (BCF) values (typically < 1.5), signifying a moderate risk of bioaccumulation. Nonetheless, the anticipated values for oral rat acute toxicity and human maximum tolerated dose exhibited considerable variation across the various substances. Collectively, these in silico findings indicate that although the active components exhibit minimal bioaccumulation potential, they may provide significant hazards concerning hepatic and cardiac toxicity, necessitating further experimental verification.

**Table 2:** Predicted oral rat acute toxicity, bioconcentration factor, and human maximum tolerated dose for the main components of QJSXP

Component	Oral Rat Acute Toxicity(LD_50_[mol/kg])	BCF(-log_10_[(mg/L)/(1000*MW)])	Human Max.tolerated dose(log_10_ mg/kg/day)
1-Methylxanthine	2.483	−0.005	1.033
2-ethyl-N-[1-[2-(1-piperidinyl)ethyl]−2-benzimidazolyl]−3-pyrazolecarboxamide	2.087	1.339	0.424
3,5-dihydroxybenzoic acid	2.227	0.34	0.918
3-Hydroxy-3-methylglutaric acid	1.511	0.1	0.407
4-Methylumbelliferone	1.981	0.652	0.356
5-(3,4-Dihydroxy-5-nitrophenyl)pentanoic acid	2.167	0.498	0.247
6-Methoxyflavone	2.019	1.53	0.077
Acacetin	2.22	1.269	0.09
Alizarin	2.255	1.805	0.154
Allopurinol	1.922	0.051	0.957
Anatoxin A	2.3	0.503	0.306
Apigenin	2.45	1.308	0.328
Baicalein	2.325	1.133	0.498
Catechol	2.14	1.331	−0.017
Daidzein	2.164	1.23	0.187
Desmethyldoxepin	2.843	1.04	0.102
Diosmetin	2.338	1.035	0.42
Ellagic acid	2.399	1.219	0.476
Eupafolin	2.419	1.007	0.544
Formononetin	1.946	1.311	0.008
Genistein	2.268	1.267	0.478
Genistin	2.643	0.465	0.412
Glutaric acid	1.622	0.19	0.547
Hispidulin	2.402	1.128	0.279
Isorhamnetin	2.407	1.098	0.576
Kaempferol	2.449	1.242	0.531
L-Phenylalanine	2.193	0.342	0.935
Luteolin	2.455	1.288	0.499
Meglutol	1.511	0.1	0.407
Nicotinamide	2.116	0.046	1.15
Nicotinic acid	2.24	0.4	0.907
Oroxylin A	2.412	1.136	−0.08
Pyrogallol	2.049	1.259	−0.269
Succinic acid	1.618	0.184	0.641
Tectorigenin	2.33	1.085	0.334
Theophylline	2.308	0.088	−0.162

## Discussion

This study thoroughly combined LC-MS, network pharmacology, MR, and molecular simulation techniques to furnish evidence for probable causative relationships between 12 principal pharmacological targets (including SYK, HIF1A, and MTOR, among others) and ITP. Molecular docking and molecular dynamics simulations corroborated that significant active chemicals found in QJSXP, including Luteolin and Ellagic acid, may form complexes with high binding affinity and preserve structural stability with these targets. The data indicate that the active components of QJSXP may influence therapeutic effects via altering the activity of these primary targets.

### Fundamental target examination

Our research revealed 12 core targets potentially implicated in the therapeutic efficacy of QJSXP against ITP: CYP1B1, SYK, CCND1, AKT1, LYN, FYN, HIF1A, BLM, HPGD, MTOR, STAT3, and ADAM10. CCND1 (Cyclin D1) is recognised for its role in regulating the G1/S phase transition of the cell cycle and facilitating cell proliferation [54]. Altered expression of CCND1 in megakaryocytes may impact their proliferation and differentiation, whereas inadequate expression could hinder the proliferation of megakaryocyte precursors [55, 56]. The translation of CCND1 relies on the activation of the mTOR signalling system; mTOR inhibition further diminishes CCND1 protein synthesis, resulting in inadequate platelet formation [57]. Research demonstrates that AKT1 is involved in the regulation of cell growth and death, with reduced AKT activity likely leading to impaired megakaryocyte maturation [58]. In megakaryocytes, the interaction of c-MPL with TPO induces c-MPL homodimerization, subsequently activating signaling pathways such as JAK/STAT, PI3K/Akt, and Ras/MAPK to regulate platelet production [59]. In monocyte-derived dendritic cells (moDCs) from ITP patients, the mTORC1 signaling pathway is markedly upregulated, resulting in the synthesis of co-stimulatory markers CD80/CD86 and pro-inflammatory cytokines IL-6 and IL-12, thus exacerbating systemic inflammation [60–62]. Furthermore, the number of Tregs is often reduced or their functionality impaired in individuals with ITP, hence increasing platelet destruction [63]. mTOR inhibitors (e.g., rapamycin) can reduce the aberrant activation of moDCs in ITP patients, enhance the release of immunosuppressive cytokines, and partially restore Treg functionality [64, 65]. SYK, LYN, and FYN are pivotal kinases in the downstream signaling cascade of the B-cell receptor (BCR), facilitating B-cell activation and autoantibody production via pathways including PI3K/AKT and NF-κB [66]. Upon binding of Fcγ receptors to their ligands, SYK is activated, resulting in the phosphorylation of immunoreceptor tyrosine-based activation motifs [66]. The heightened expression and activity of SYK in macrophages from ITP patients, coupled with its phosphorylation, induce cytoskeletal reorganisation, ultimately leading to the phagocytosis of antibody-coated platelets [67]. SYK inhibitors, such as fostamatinib, have been authorised for the treatment of ITP by obstructing FcγR signalling and diminishing platelet degradation [68, 69]. LYN phosphorylates CD19, thereby augmenting BCR signalling efficacy, and collectively, they synergistically modulate B cell activation, survival, and humoral immune responses [70–72]. Inhibition of LCK/FYN diminishes STAT3 phosphorylation, resulting in compromised Th17 differentiation and a transition towards Treg cells [73]. ADAM10 is present on the platelet surface and is involved in the shedding of proteins associated with platelet activation, such as GPVI and P-selectin. Dominant-negative mutations in ADAM10 identified in ITP patients may hinder T-cell development [74, 75]. Furthermore, targeted ablation of ADAM10 in T cells results in significantly less antigen-induced T cell proliferation [76].

In conclusion, QJSXP may provide therapeutic benefits for ITP by acting on multiple targets synergistically. This process enhances megakaryocyte proliferation and differentiation through the mTOR, CCND1, and AKT1 pathways, resulting in increased platelet production. Simultaneously, it diminishes FcγR-mediated platelet phagocytosis and destruction through the targeting of the SYK pathway. This drug concurrently affects mTORC1 and ADAM10-related pathways through kinases, including SYK, LYN, and FYN, thus modulating B-cell immune responses and T-cell differentiation. The synergistic effects promote megakaryocyte proliferation and differentiation, inhibit platelet clearance, decrease autoantibody synthesis, and restore T-cell homeostasis, thereby alleviating ITP symptoms and delaying disease progression.

### Biological enrichment assessment

GO functional enrichment study indicated that the biological processes influenced by QJSXP in ITP treatment encompass immune cell proliferation and differentiation, regulation of immune response, and modulation of inflammatory response, among others. These processes encompass molecular functions like phosphate metabolism, cellular motility, protein modification, and enzyme-linked receptor functioning. The KEGG pathway enrichment study indicated that the signalling pathways predominantly associated with QJSXP’s involvement in ITP pertain to immune response, cell proliferation and differentiation, and inflammation. Aberrant proliferation, metabolism, and survival of immune cells directly contribute to immunological dysregulation, a crucial element in the pathogenesis of ITP. The PI3K/Akt/mTOR pathway is activated in inflammatory circumstances and is essential for immune control by influencing several immune cell activities [77–81]. AKT1 facilitates the phosphorylation of BAD, a Bcl-2 family member, inhibiting its pro-apoptotic function and liberating the anti-apoptotic protein Bcl-XL, which safeguards B cells from apoptosis and may worsen thrombocytopenia [82–86]. In contrast, the binding of platelets to anti-GPIbα antibodies can activate Akt, which in turn activates downstream protein kinase A and pro-apoptotic proteins, facilitating calcium mobilisation and so aiding in platelet clearance [87, 88]. Inhibition of Akt expression can attenuate apoptotic signals and safeguard platelets from elimination [88]. Anti-αvβ3 integrin autoantibodies are commonly identified in patients with chronic ITP, as they can impede megakaryocyte migration by inhibiting Akt phosphorylation, thereby exacerbating platelet depletion [89]. The therapeutic efficacy of TPO-RAs in ITP is somewhat facilitated by PI3K/Akt signaling, resulting in elevated platelet counts [90, 91]. The MAPK signaling pathway, specifically JNK, p38, and ERK, regulates essential cellular activities and, in T cells, facilitates activation, Th1/Th2 differentiation, and the synthesis of IFN-γ, IL-2, and IL-4, thus influencing adaptive immune responses [92–95]. Moreover, the MAPK signalling pathway is crucial in platelet activation and aggregation [96–98]. Hypoxia-inducible factor-1α (HIF-1α) is a key transcription factor in cellular responses to hypoxia, closely associated with the regulation of hematopoietic stem cells (HSCs) and essential for megakaryocyte growth, and it modulates CD8 + T cell involvement in immune responses, and studies indicate reduced HIF-1α expression in ITP [99–102]. The JAK/STAT pathway plays a critical role in cell proliferation, differentiation, and immune regulation. Previous studies demonstrate that the JAK-STAT signaling pathway is active in individuals with ITP, likely influencing Th1, Th2, and related cytokine production [103–105]. Research has identified markedly reduced expression levels of six miRNAs in the peripheral blood of ITP patients, which impacts Th1 and Th2 cell development and contributes to immune system dysregulation in these individuals [106].

### Examination of potential active constituents

This study initially tested and discovered probable active components of QJSXP pertinent to ITP treatment, including Luteolin, Ellagic acid, Kaempferol, Apigenin, and Eupafolin, among others. Preclinical investigations indicate that Luteolin can sustain steady circulatory function via various processes, including enhancing procoagulant substance levels, stimulating the gene production of coagulation factors, and increasing coagulation factor concentrations. Experimental investigations indicate that Kaempferol, Luteolin, and Apigenin exhibit antioxidant qualities and can impede platelet aggregation and adhesion, hence diminishing thrombus formation, while also influencing platelet activation [107]. Luteolin also inhibits the TNF-α-induced upregulation of JAK2 and STAT3 mRNA expression through the JAK/STAT pathway. The inhibitory effect exhibits a positive correlation with drug concentration and is closely linked to the regulation of inflammation [108, 109]. Apigenin exhibits various effects in in vitro and in vivo studies, such as cell cycle arrest, induction of apoptosis, anti-inflammatory activity, and antioxidant properties [110]. Kaempferol suppresses the activation of HIF-1α and VEGFR in endothelial cells through the ERK/MAPK and PI3K/AKT/mTOR signalling pathways, thereby impeding angiogenesis [111]. Numerous experimental studies have demonstrated that Kaempferol can impede the phosphorylation of PI3K and Akt, thereby safeguarding cells from the repercussions of inflammatory cytokine activation [112–114]. Kaempferol can efficiently suppress M1 polarisation of macrophages through the MAPK/NF-κB signalling pathway, hence enhancing immunological and inflammatory responses [115]. Ellagic acid has the capacity to mitigate or avert in vivo toxicity via various mechanisms, including the inhibition of nitric oxide production, the blockade of NF-κB activation, and the augmentation of cellular antioxidant systems [116, 117]. Eupafolin demonstrates several pharmacological properties, encompassing anti-inflammatory, anti-cancer, antioxidant, anti-allergic, cardioprotective, and neuroprotective effects. It exerts its anti-tumour impact by downregulating MMP9 expression through the FAK/PI3K/AKT pathway and rectifies Th1/Th2 imbalance by blocking the p38 MAPK signalling pathway. Eupafolin can downregulate the expression of TNF-α, IL-1β, and IL-6 in cells and inhibit the phosphorylation of the JAK2/STAT3 signalling pathway, hence obstructing the nuclear translocation of p-STAT3 to mitigate inflammation [118–120]. The active components may also contribute to platelet formation. Research on ellagic acid and its derivatives has demonstrated their ability to enhance the proliferation of haematopoietic progenitor cells and the differentiation of megakaryocytes, consequently promoting platelet formation [121, 122]. In murine studies, genistin modulates megakaryocyte development through the activation of PI3K/AKT and MEK/ERK pathways via ERβ. It largely improves platelet function and expedites platelet recovery during thrombocytopenia, without elevating platelet count in normal mice [123]. In vitro experiments, the duration for platelet recovery was reduced in single umbilical cord blood units expanded ex vivo with nicotinamide [124]. A recent Phase III clinical research study shown that the utilisation of nicotinamide-based expansion technology during haematopoietic stem cell transplantation enhances platelet reconstitution [125]. Oroxylin A suppresses platelet activation in a concentration-dependent manner through the GPVI signalling pathway by mitigating oxidative stress and binding to SHP-2, without exhibiting considerable pharmacological toxicity [126]. Wedelolactone modulates mitochondrial function to meet the energy requirements of megakaryocyte differentiation and markedly enhances the differentiation of megakaryocytes and the production of HSCs, megakaryocytes, and reticulated platelets, engaging the AMPK and MAPK signalling pathways [127]. Molecular docking experiments demonstrated a robust binding affinity between several principal chemicals from QJSXP (including Luteolin, Ellagic acid, Kaempferol, Apigenin, and Eupafolin, among others) and critical target proteins such as CYP1B1, SYK, FYN, and STAT3. This offers computational validation for the dependability of the network prediction outcomes. Molecular dynamics simulations further confirmed the stable binding of the principal active components to their target proteins. These compounds influence the survival, differentiation, signalling pathways, and inflammatory responses of immune cells, such as B cells, T cells, and megakaryocytes, thereby contributing to the pathogenesis of ITP. They particularly exhibit synergistic effects in rectifying apoptosis imbalance, abnormal activation of signalling pathways, the establishment of a pro-inflammatory microenvironment, and platelet irregularities.

### Analysis of component toxicity

Toxicology predictions for the constituents of QJSXP suggest that although some compounds exhibit theoretical toxicity risks, these potential toxicities are generally not significant within probable pharmacological dose ranges, and the notable therapeutic effects usually surpass potential adverse effects. The prediction model indicated that the components 3,5-dihydroxybenzoic acid, Baicalein, Luteolin, and Apigenin demonstrated heightened projected harmful effects. 3,5-Dihydroxybenzoic acid exhibited a significant potential risk for nephrotoxicity, as well as possible toxicity to the BBB and clinical damage. The anticipated hazards for Baicalein and Luteolin were chiefly associated with respiratory toxicity, nephrotoxicity, and carcinogenicity. Nonetheless, in vivo investigations have indicated no considerable toxicity following prolonged oral treatment of Baicalein in rats (40–80 mg/kg/d for 10 weeks) [128]. Phase I clinical trial data demonstrate that healthy volunteers administered single oral doses of 100–2800 mg Baicalein chewable tablets did not experience any serious adverse events [129]. Only 11 cases exhibited mild gastrointestinal discomfort, which resolved spontaneously without specific intervention, and there was no evidence of hepatotoxicity or nephrotoxicity [129]. Prolonged oral administration of Luteolin in rats (30 mg/kg for 20 days) exhibited no discernible alterations in body weight or organ damage [130]. Apigenin generally demonstrated safety across most toxicity categories; nevertheless, possible concerns were anticipated for respiratory, renal, and ecotoxicity. The assessment of acute toxicity of Apigenin revealed no fatalities or symptoms of toxicity in mice or rats at oral doses up to 5000 mg/kg [131]. In vitro carcinogenicity evaluations revealed no toxicity or mutagenesis effects associated with Apigenin [132]. The in vitro haemolytic activity of Apigenin after 30 min of treatment was below the tolerable threshold of 5%, indicating possible safety for intravenous administration levels [133]. A single intraperitoneal administration of 100 and 200 mg/kg Apigenin in male Swiss mice induced oxidative stress and resultant hepatic injury [134]. Although several studies have indicated safety problems regarding Apigenin, these issues may pertain to the method of administration, and no research has identified safety risks linked to oral consumption of Apigenin. In toxicological predictions, many component toxicity endpoints concentrate on nephrotoxicity, respiratory toxicity, and the BBB. Allopurinol, in comparison to other ingredients, has the potential to cause acute allergic-like kidney injury, primarily presenting as acute interstitial nephritis and vasculitis phenotypes [135, 136]. Certain phenolic compounds are implicated in regulating cell proliferation and apoptosis. Research indicates that pyrogallol inhibits proliferation of mouse As4.1 periglomerular cells by inducing G2 phase cell cycle arrest and apoptosis [137]. Moreover, pyrogallol can modulate the first relaxation of peribronchial cells. In research involving human lung cancer Calu-6 cells, pyrogallol triggers apoptosis in isolated lung cells by inducing G2-specific cell cycle arrest [138–140]. Theophylline is capable of traversing the BBB and may not be regulated by P-glycoprotein [141]. Hispidulin can traverse the BBB and demonstrates anticonvulsant and antiepileptic properties [142, 143]. High-dose nicotinamide is regarded as a potentially hazardous substance when given to adults at dosages over 3 g per day [144]. Elevated dosages of nicotinic acid, especially sustained-release formulations, have been shown to promote drug-related liver harm, with histological analysis indicating widespread microcystic steatosis [145]. Our study indicates that the predicted Oral Rat Acute Toxicity (LD₅₀), BCF, and Human Maximum Tolerated Dose (HMTD) for the primary QJSXP components (Table 2) collectively imply low-to-moderate acute toxicity, restricted bioaccumulation potential, and dose ranges suitable for potential clinical exposure, necessitating further experimental validation. Corticosteroids are the primary treatment for ITP; however, prolonged usage is linked to cumulative side effects [11]. TPO-RAs serve as a second-line treatment. Eltrombopag possesses a hepatotoxicity warning; hence, liver function must be meticulously monitored throughout the treatment [146, 147]. Romiplostim has been associated with elevated levels of bone marrow fibrosis in certain people [148]. Moreover, chemicals originating from natural sources typically demonstrate elevated safety profiles, including isoflavones and flavonoids such as Genistein and Luteolin. In contrast to traditional ITP therapies, the proposed constituents of QJSXP are commonly found in food or utilized in Traditional Chinese Medicine; their prolonged use offers supportive evidence for a favorable safety profile in clinical application, although formal pharmacological and toxicological validation is still required [149, 150]. Research indicates that optimising metabolic properties, such as enhancing bioavailability or altering molecular structures, can improve drug efficacy and mitigate toxicity risks [151, 152]. In actual practice, Traditional Chinese Medicine (TCM) is not merely a compilation of individual substances; rather, the synergistic combination of various herbs can enhance their benefits and alleviate their limits, demonstrating more efficacy in disease treatment compared to singular medications [153]. A favourable equilibrium between toxicity and therapeutic efficacy of the components is evident, especially within appropriate dosage levels, where potential adverse effects are typically acceptable and controlled.

## Conclusion

This study employed a comprehensive methodology that included network pharmacology, Mendelian randomisation analysis of pharmaceutical targets, molecular docking, and molecular dynamics simulations. Our research indicates that the principal active constituents of QJSXP, including Luteolin, Ellagic acid, Kaempferol, Apigenin, and Eupafolin, may influence ITP by modulating signalling pathways such as Pathways in Cancer, the PI3K-Akt signalling pathway, MicroRNAs in cancer, and the MAPK signalling pathway, potentially via targets such as CYP1B1, SYK, FYN, and STAT3, as determined through target prediction, protein interaction network construction, and GO/KEGG pathway enrichment analysis. Network pharmacology fundamentally depends on network databases and pre-existing experimental data for its modelling. The diversity in original experimental data and spectral information obtained under diverse experimental settings results in these approaches possessing an intrinsic rate of false positives and false negatives [154]. This work largely relies on computer simulations and literature analysis to forecast and elucidate the mechanisms through which QJSXP influences ITP. Consequently, our findings may reveal inconsistencies when compared to established databases or particular experimental data.

We expect these analytical results to offer a theoretical foundation and directed guidance for future in-depth investigation. Our study team will persist in examining the pharmacological mechanisms of QJSXP and intends to perform additional animal or cellular tests to elucidate the exact targets and molecular mechanisms involved in its intervention in ITP.

## Supplementary Information

Supplementary Material 1.

Supplementary Material 2.

Supplementary Material 3.

Supplementary Material 4.

Supplementary Material 5.

Supplementary Material 6.

Supplementary Material 7.

Supplementary Material 8.

Supplementary Material 9.

Supplementary Material 10.

Supplementary Material 11.

Supplementary Material 12.

Supplementary Material 13.

## Data Availability

The datasets generated and analysed during the current study are included in this published article and its supplementary information files. The raw data supporting the findings of this study were primarily derived from publicly available databases, including PubChem (https://pubchem.ncbi.nlm.nih.gov), SwissTargetPrediction (http://swisstargetprediction.ch), PharmMapper (http://www.lilab-ecust.cn/pharmmapper), SuperPred (https://prediction.charite.de), GeneCards (https://www.genecards.org), OMIM (https://www.omim.org), DRUGBANK (https://go.drugbank.com), GAD (https://maayanlab.cloud/Harmonizome/resource/Genetic+Association+Database), DisGeNET (https://disgenet.com), HERB (http://herb.ac.cn), UniProt (https://www.uniprot.org), STRING (https://cn.string-db.org), Metascape (https://metascape.org/gp/index.html), eQTLgen (https://eqtlgen.org), the GWAS database (EBI GWAS Catalog, https://www.ebi.ac.uk/gwas), RCSB PDB (https://www.rcsb.org), and ProTox (https://tox.charite.de/protox3). All original URLs are provided in the text.

## Abbreviations

QJSXP: Qian Ji Sheng Xue Pian
ITP: Primary immune thrombocytopenia
LC-MS: Liquid chromatography-mass spectrometry
MR: Mendelian randomization
TPO-RAs: Thrombopoietin receptor agonists
TCM: Traditional Chinese Medicine
ESI: Electrospray ionization
GO: Gene ontology
BP: Biological process
CC: Cellular component
MF: Molecular function
KEGG: Kyoto encyclopaedia of genes and genomes
IVW: Inverse variance weighted
FDR: False discovery rate
RMSD: Root mean square deviation
RMSF: Root mean square fluctuation
Rg: Radius of gyration
SASA: Solvent accessible surface area
OB: Oral bioavailability
DL: Drug-likeness
EF: Enrichment factors
BCF: Bioconcentration factor
moDCs: Monocyte-derived dendritic cells
