# Supplementary material for: Multimethodological and multiscale investigation of the therapeutic mechanism of Qian Ji Sheng Xue Pian in treating primary immune thrombocytopenia
Source: Hereditas. 2025 Dec 6;163:11. doi: 10.1186/s41065-025-00620-3 (PMC12797464; doi:10.1186/s41065-025-00620-3)
Supplement: Supplementary file 1 — Supplementary Material 1. [file 41065_2025_620_MOESM1_ESM.docx]

Supplementary Figures:

Figure S1: Total Ion Chromatograms (TICs) of QJSXP extract. The left panel, designated "POS," displays the TIC obtained in positive ion mode. The right panel, designated "NEG," displays the TIC obtained in negative ion mode. The x-axis denotes retention period (period, min), whereas the y-axis indicates relative ion abundance (relative abundance). The chromatograms depict the complexity and peak distribution of the chemical constituents identified in the QJSXP extract at varying ionization settings by LC-MS. Peaks represent distinct or co-eluting chemicals.

Figure S2: Detailed ESI-MS spectra of potential active components in QJSXP. a:Desmethyldoxepin(1)；b:Meglutol(2)；c:Nicotinamide(3)；d：Nicotinic acid(4);e:L-Phenylalanine(5);f:3,5-dihydroxybenzoic acid(6);g:5-(3,4-Dihydroxy-5-nitrophenyl)pentanoic acid(7)；h：Anatoxin A(8)；i：4-Methylumbelliferone(9)；j：Genistein(10)；k：Diosmetin(11)；l：Genistin(12)；m：Luteolin(13)；n：Kaempferol(14)；o：Tectorigenin(15)；p：Isorhamnetin(16)；q：Baicalein(17)；r：Oroxylin A(18)；s：6-Methoxyflavone(19)；t：2-ethyl-N-[1-[2-(1-piperidinyl)ethyl]-2-benzimidazolyl]-3-pyrazolecarboxamide(20)；u：Theophylline(21)；v：1-Methylxanthine(22)；w：Allopurinol(23)；x：Glutaric acid(24)；y：Succinic acid(25)；z：3-Hydroxy-3-methylglutaric acid(26)；aa：Pyrogallol(27)；ab：Catechol(28)；ac：Ellagic acid(29)；ad：Luteolin(13)；ae：Acacetin(30)；af：Wedelolactone(31)；ag：Apigenin(32)；ah：Kaempferol(14)；ai：Hispidulin(33)；aj：Eupafolin(34)；ak：Formononetin(35)；al：Alizarin(36)；am：Daidzein(37).

Figure S3: KEGG Visual Pathway Details Diagram.(a)Pathways in cancer; (b)PI3K-Akt signaling pathway; (c)MicroRNAs in cancer ;(d)MAPK signaling pathway; (e)JAK-STAT signaling pathway

Figure S4: a:This bar chart illustrates the outcomes of a redocking benchmark.The X-axis denotes each frame or conformation derived from the simulation or docking procedure. The Y-axis displays the RMSD in Angstroms (Å) for each frame. RMSD quantifies the mean distance between corresponding atoms of two structures. In this context, it presumably quantifies the deviation of each docked pose from a recognized reference structure (e.g., the crystal structure). The blue bars denote the RMSD value corresponding to each individual frame. Reduced bar heights signify a superior alignment with the reference structure.The red dashed line is positioned at 2.0 Å, serving as a standard benchmark for successful redocking. Frames exhibiting an RMSD below this threshold are typically regarded as successfully redocked, indicating that the predicted conformation closely resembles the reference structure.The data indicates that the majority of the frames have an RMSD well below the 2.0 Å threshold, suggesting that the redocking process was predominantly effective in replicating the reference structure. b,c:The redocking RMSD of the co-crystallization ligands was 1.6 Å and 1.8 Å, respectively, both below 2.0 Å. d: Time-dependent RMSD trajectories of the protein backbone during molecular dynamics simulations, contrasting the control medication alisertib (blue) with the plant chemical (orange). e: RMSD curves of heavy atoms in the ligand throughout simulations, comparing the control medication and the plant chemical. f: The picture presents a comparative plot of RMSF for each residue. The blue curve denotes the Control group. The orange curve denotes the Phytochemical group. The x-axis represents residue numbers, the y-axis denotes RMSF (Å), and the gray shaded area indicates the binding pocket residue region. g: The left bar chart compares the overall binding free energy (ΔG_bind) data, with blue denoting the control ligand (alisertib) and orange indicating the phytochemical. Both demonstrate ΔG_bind values approximately between -20 and -25 kcal/mol, signifying a robust binding affinity. The right bar chart illustrates the findings of energy decomposition, including van der Waals interactions, electrostatic interactions, polar solvation, and nonpolar solvation. The colors blue and orange persist in symbolizing the control ligand and phytochemical, respectively. Van der Waals and electrostatic interactions serve as the principal stabilizing forces.

Figure S5: Comprehensive molecular docking analyses of QJSXP active constituents with core risk gene products. (a):Genistin docking CYP1B1; (b)Genistein docking CYP1B1; (c):Luteolin docking SYK; (d):Eupafolin docking SYK; (e):Luteolin docking CCND1; (f):Ellagic acid docking CCND1; (g):1-Methylxanthine docking AKT1; (h):Alizarin docking AKT1; (i):Theophylline docking LYN; (j):Apigenin docking LYN; (k):Anatoxin A docking FYN; (l):Pyrogallol docking FYN; (m):Anatoxin A docking HIF1A; (n):Theophylline docking HIF1A; (o)Desmethyldoxepin docking BLM; (p):Hispidulin docking BLM; (q):4-Methylumbelliferone docking HPGD; (r)Isorhamnetin docking HPGD; (s):Theophylline docking MTOR; (t):4-Methylumbelliferone docking MTOR; (u):1-Methylxanthine docking STAT3; (v):Ellagic acid docking STAT3; (w):Apigenin docking ADAM10; (x):Luteolin docking ADAM10.
