# Supplementary material for: Multimethodological and multiscale investigation of the therapeutic mechanism of Qian Ji Sheng Xue Pian in treating primary immune thrombocytopenia
Source: Hereditas. 2025 Dec 6;163:11. doi: 10.1186/s41065-025-00620-3 (PMC12797464; doi:10.1186/s41065-025-00620-3)
Supplement: Supplementary file 2 — Supplementary Material 2. [file 41065_2025_620_MOESM2_ESM.docx]

Supplementary Tables:

Table S1: Identification of Partial Potential Bioactive Compounds in QJSXP by LC-MS and Their Mass Spectral Attributes.

Table S2: Enrichment results of targets and pathways for regulating ITP by QJSXP.

Table S3: Defined parameters of network node attributes for the principal active components of QJSXP.
