# Supplementary material for: Multimethodological and multiscale investigation of the therapeutic mechanism of Qian Ji Sheng Xue Pian in treating primary immune thrombocytopenia
Source: Hereditas. 2025 Dec 6;163:11. doi: 10.1186/s41065-025-00620-3 (PMC12797464; doi:10.1186/s41065-025-00620-3)
Supplement: Supplementary file 3 — Supplementary Material 3. [file 41065_2025_620_MOESM3_ESM.docx]

| Mode | Name | Formula | PPM | Calc. MW | m/z | RT [min] | Reference Ion |
| --- | --- | --- | --- | --- | --- | --- | --- |
| ESI+ | Desmethyldoxepin | C18 H19 N O | 3.18 | 265.15184 | 266.15912 | 0.82 | [M+H]+1 |
| ESI+ | Meglutol | C6 H10 O5 | -3.71 | 162.05222 | 195.08572 | 0.89 | [M+H+MeOH]+1 |
| ESI+ | Nicotinamide | C6 H6 N2 O | -4.63 | 122.04745 | 123.05492 | 1.26 | [M+H]+1 |
| ESI+ | Nicotinic acid | C6 H5 N O2 | -2.97 | 123.03166 | 124.03893 | 1.27 | [M+H]+1 |
| ESI+ | L-Phenylalanine | C9 H11 N O2 | -2.76 | 165.07852 | 166.0858 | 3.17 | [M+H]+1 |
| ESI+ | 3,5-dihydroxybenzoic acid | C7 H6 O4 | -2.27 | 154.02626 | 155.03355 | 3.89 | [M+H]+1 |
| ESI+ | 5-(3,4-Dihydroxy-5-nitrophenyl)pentanoic acid | C11 H13 N O6 | -1.65 | 255.07387 | 238.07069 | 4.53 | [M+H-H2O]+1 |
| ESI+ | Anatoxin A | C10 H15 N O | -2.53 | 165.11495 | 166.12222 | 6.04 | [M+H]+1 |
| ESI+ | 4-Methylumbelliferone | C10 H8 O3 | -2.92 | 176.04683 | 209.08029 | 6.23 | [M+H+MeOH]+1 |
| ESI+ | Genistein | C15 H10 O5 | -1.96 | 270.05229 | 271.05957 | 8.66 | [M+H]+1 |
| ESI+ | Diosmetin | C16 H12 O6 | -3.58 | 318.07312 | 301.06998 | 8.85 | [M+H-H2O]+1 |
| ESI+ | Genistin | C21 H20 O10 | -3.34 | 432.1042 | 433.11148 | 10.06 | [M+H]+1 |
| ESI+ | Luteolin | C15 H10 O6 | -2.96 | 286.04689 | 287.05417 | 10.23 | [M+H]+1 |
| ESI+ | Kaempferol | C15 H10 O6 | -3.17 | 286.04683 | 287.05411 | 11.43 | [M+H]+1 |
| ESI+ | Tectorigenin | C16 H12 O6 | -3.11 | 300.06246 | 301.06973 | 11.50 | [M+H]+1 |
| ESI+ | Isorhamnetin | C16 H12 O7 | -3.37 | 316.05724 | 317.06451 | 11.67 | [M+H]+1 |
| ESI+ | Baicalein | C15 H10 O5 | -2.8 | 270.05207 | 271.05936 | 13.18 | [M+H]+1 |
| ESI+ | Oroxylin A | C16 H12 O5 | -4.75 | 284.06713 | 285.0744 | 13.99 | [M+H]+1 |
| ESI+ | 6-Methoxyflavone | C16 H12 O3 | -2.77 | 252.07795 | 285.11142 | 20.9 | [M+H+MeOH]+1 |
| ESI+ | 2-ethyl-N-[1-[2-(1-piperidinyl)ethyl]-2-benzimidazolyl]-3-pyrazolecarboxamide | C20 H26 N6 O | 4.29 | 366.21838 | 367.22984 | 24.76 | [M+H]+1 |
| ESI- | Theophylline | C7 H8 N4 O2 | -1.11 | 180.06316 | 179.05602 | 0.87 | [M-H]-1 |
| ESI- | 1-Methylxanthine | C6 H6 N4 O2 | -8.12 | 166.04773 | 165.04045 | 0.88 | [M-H]-1 |
| ESI- | Allopurinol | C5 H4 N4 O | -8.86 | 136.03731 | 135.03003 | 0.88 | [M-H]-1 |
| ESI- | Glutaric acid | C5 H8 O4 | 0.9 | 132.04238 | 131.03511 | 0.89 | [M-H]-1 |
| ESI- | Succinic acid | C4 H6 O4 | 0.59 | 118.02668 | 117.0194 | 1.57 | [M-H]-1 |
| ESI- | 3-Hydroxy-3-methylglutaric acid | C6 H10 O5 | -0.56 | 162.05273 | 161.04546 | 1.78 | [M-H]-1 |
| ESI- | Pyrogallol | C6 H6 O3 | -0.03 | 126.03169 | 125.02441 | 1.89 | [M-H]-1 |
| ESI- | Catechol | C6 H6 O2 | 0.76 | 110.03686 | 109.02959 | 4.96 | [M-H]-1 |
| ESI- | Ellagic acid | C14 H6 O8 | -4.64 | 302.00487 | 300.99759 | 7.66 | [M-H]-1 |
| ESI- | Luteolin | C15 H10 O6 | -3.29 | 286.0468 | 285.03952 | 10.23 | [M-H]-1 |
| ESI- | Acacetin | C16 H12 O5 | -3.36 | 284.06752 | 283.06024 | 10.33 | [M-H]-1 |
| ESI- | Wedelolactone | C16 H10 O7 | -3.61 | 314.04152 | 313.03424 | 10.52 | [M-H]-1 |
| ESI- | Apigenin | C15 H10 O5 | -3.1 | 270.05198 | 269.04471 | 11.23 | [M-H]-1 |
| ESI- | Kaempferol | C15 H10 O6 | -3.61 | 286.04671 | 285.03943 | 11.41 | [M-H]-1 |
| ESI- | Hispidulin | C16 H12 O6 | -3.32 | 300.06239 | 299.05511 | 11.49 | [M-H]-1 |
| ESI- | Eupafolin | C16 H12 O7 | -2.99 | 316.05736 | 315.05008 | 11.67 | [M-H]-1 |
| ESI- | Formononetin | C16 H12 O4 | -2.61 | 268.07286 | 267.06558 | 12.63 | [M-H]-1 |
| ESI- | Alizarin | C14 H8 O4 | -2.45 | 240.04167 | 239.03439 | 14.13 | [M-H]-1 |
| ESI- | Daidzein | C15 H10 O4 | -2.78 | 254.0572 | 253.04993 | 15.94 | [M-H]-1 |

**Table S1 Identification of Partial Potential Bioactive Compounds in QJSXP by LC-MS and Their Mass Spectral Attributes**
