# Supplementary material for: Multimethodological and multiscale investigation of the therapeutic mechanism of Qian Ji Sheng Xue Pian in treating primary immune thrombocytopenia
Source: Hereditas. 2025 Dec 6;163:11. doi: 10.1186/s41065-025-00620-3 (PMC12797464; doi:10.1186/s41065-025-00620-3)
Supplement: Supplementary file 4 — Supplementary Material 4. [file 41065_2025_620_MOESM4_ESM.docx]

| **Table S2 Enrichment results of targets and pathways for regulating/treating ITP by QJSXP** | | | | |
| --- | --- | --- | --- | --- |
| **GO** | **Description** | **LogP** | **Count** | **Hits** |
| hsa05200 | Pathways in cancer | -35.2251 | 32 | AKT1、AR、CCND1、BCL2、BRAF、CDK4、CDK6、EGFR、ERBB2、ESR1、F2、FGFR1、FLT3、MTOR、HIF1A、IL2、SMAD3、MET、MMP2、MMP9、NFE2L2、NFKB1、NOS2、PDGFRA、PDGFRB、PIK3CD、MAPK1、STAT1、STAT3、TGFBR2、TP53、CXCR4 |
| hsa04151 | PI3K-Akt signaling pathway | -23.8415 | 22 | AKT1、CCND1、BCL2、CDK4、CDK6、EGFR、ERBB2、FGFR1、FLT3、MTOR、IL2、KDR、MET、NFKB1、PDGFRA、PDGFRB、PIK3CD、PIK3CG、MAPK1、SYK、TEK、TP53 |
| hsa05206 | MicroRNAs in cancer | -19.0186 | 18 | CCND1、BCL2、CDK6、CYP1B1、EGFR、ERBB2、MTOR、MET、MMP9、ABCC1、NFKB1、PDGFRA、PDGFRB、ABCB1、PIK3CD、MAPK1、STAT3、TP53 |
| hsa04010 | MAPK signaling pathway | -16.3515 | 16 | AKT1、BRAF、EGFR、ERBB2、FGFR1、FLT3、KDR、MAPT、MET、NFKB1、PDGFRA、PDGFRB、MAPK1、TEK、TGFBR2、TP53 |
| hsa04630 | JAK-STAT signaling pathway | -15.4104 | 13 | AKT1、CCND1、BCL2、EGFR、MTOR、IL2、PDGFRA、PDGFRB、PIK3CD、PTPN11、STAT1、STAT3、THPO |
| hsa04014 | Ras signaling pathway | -13.4418 | 13 | AKT1、EGFR、FGFR1、FLT3、KDR、MET、NFKB1、PDGFRA、PDGFRB、PIK3CD、MAPK1、PTPN11、TEK |
| hsa05220 | Chronic myeloid leukemia | -18.0278 | 12 | AKT1、CCND1、BRAF、CDK4、CDK6、SMAD3、NFKB1、PIK3CD、MAPK1、PTPN11、TGFBR2、TP53 |
| hsa04072 | Phospholipase D signaling pathway | -14.4548 | 12 | AKT1、EGFR、F2、MTOR、FYN、PDGFRA、PDGFRB、PIK3CD、PIK3CG、MAPK1、PTPN11、SYK |
| hsa04015 | Rap1 signaling pathway | -12.6115 | 12 | AKT1、BRAF、EGFR、FGFR1、KDR、MET、PDGFRA、PDGFRB、PIK3CD、MAPK1、SRC、TEK |
| hsa04066 | HIF-1 signaling pathway | -16.1246 | 12 | AKT1、BCL2、EGFR、ERBB2、MTOR、HIF1A、NFKB1、NOS2、PIK3CD、MAPK1、STAT3、TEK |
| hsa04218 | Cellular senescence | -12.6036 | 11 | AKT1、CCND1、CDK4、CDK6、MTOR、SMAD3、NFKB1、PIK3CD、MAPK1、TGFBR2、TP53 |
| hsa04810 | Regulation of actin cytoskeleton | -10.7928 | 11 | AKT1、BRAF、EGFR、F2、FGFR1、PDGFRA、PDGFRB、PIK3CD、MAPK1、SRC、CXCR4 |
| hsa04611 | Platelet activation | -12.0707 | 10 | AKT1、F2、FYN、LYN、PIK3CD、PIK3CG、MAPK1、PTGS1、SRC、SYK |
| hsa04926 | Relaxin signaling pathway | -11.8984 | 10 | AKT1、EGFR、MMP2、MMP9、NFKB1、NOS2、PIK3CD、MAPK1、SRC、TGFBR2 |
| hsa04068 | FoxO signaling pathway | -10.2439 | 9 | AKT1、CCND1、BRAF、EGFR、SMAD3、PIK3CD、MAPK1、STAT3、TGFBR2 |
| hsa04020 | Calcium signaling pathway | -7.7634 | 9 | EGFR、ERBB2、FGFR1、KDR、MET、NOS2、PDGFRA、PDGFRB、CXCR4 |
| hsa04625 | C-type lectin receptor signaling pathway | -11.1762 | 9 | AKT1、IL2、NFKB1、PIK3CD、MAPK1、PTPN11、SRC、STAT1、SYK |
| hsa04659 | Th17 cell differentiation | -11.0643 | 9 | MTOR、HIF1A、IL2、SMAD3、NFKB1、MAPK1、STAT1、STAT3、TGFBR2 |
| hsa04012 | ErbB signaling pathway | -10.2706 | 8 | AKT1、BRAF、EGFR、ERBB2、MTOR、PIK3CD、MAPK1、SRC |
| hsa04660 | T cell receptor signaling pathway | -9.0434 | 8 | AKT1、CDK4、FYN、IL2、NFKB1、PIK3CD、MAPK1、PTPN11 |
