# Supplementary material for: Multimethodological and multiscale investigation of the therapeutic mechanism of Qian Ji Sheng Xue Pian in treating primary immune thrombocytopenia
Source: Hereditas. 2025 Dec 6;163:11. doi: 10.1186/s41065-025-00620-3 (PMC12797464; doi:10.1186/s41065-025-00620-3)
Supplement: Supplementary file 5 — Supplementary Material 5. [file 41065_2025_620_MOESM5_ESM.docx]

| **Table S3 Defined Parameters of Network Node Attributes for the Principal Active Components of QJSXP** | | | | |
| --- | --- | --- | --- | --- |
| **Number** | **Name** | **Degree** | **Betweenness Centrality** | **Closeness Centrality** |
| QJ46 | Luteolin | 84 | 0.0773 | 0.4095 |
| QJ32 | Ellagic acid | 83 | 0.1509 | 0.4095 |
| QJ44 | Kaempferol | 73 | 0.0797 | 0.3982 |
| QJ22 | Apigenin | 72 | 0.0603 | 0.3945 |
| QJ34 | Eupafolin | 62 | 0.0336 | 0.3917 |
| QJ11 | 5-(3,4-Dihydroxy-5-nitrophenyl)pentanoic acid | 60 | 0.0899 | 0.3917 |
| QJ39 | Genistin | 59 | 0.0418 | 0.3910 |
| QJ2 | Wedelolactone | 56 | 0.0291 | 0.3854 |
| QJ31 | Diosmetin | 56 | 0.0270 | 0.3881 |
| QJ38 | Genistein | 54 | 0.0258 | 0.3840 |
| QJ43 | Isorhamnetin | 52 | 0.0202 | 0.3861 |
| QJ18 | Acacetin | 47 | 0.0142 | 0.3773 |
| QJ41 | Hispidulin | 47 | 0.0126 | 0.3760 |
| QJ29 | Daidzein | 46 | 0.0181 | 0.3780 |
| QJ23 | Baicalein | 43 | 0.0311 | 0.3760 |
| QJ30 | Desmethyldoxepin | 43 | 0.1103 | 0.3780 |
| QJ25 | Biochanin A | 39 | 0.0140 | 0.3727 |
| QJ64 | Tectorigenin | 38 | 0.0132 | 0.3740 |
| QJ33 | Emodin | 35 | 0.0154 | 0.3708 |
| QJ47 | Meglutol | 34 | 0.0273 | 0.3715 |
