# Supplementary figures and images for: Multimethodological and multiscale investigation of the therapeutic mechanism of Qian Ji Sheng Xue Pian in treating primary immune thrombocytopenia
Source: Hereditas. 2025 Dec 6;163:11. doi: 10.1186/s41065-025-00620-3 (PMC12797464; doi:10.1186/s41065-025-00620-3)

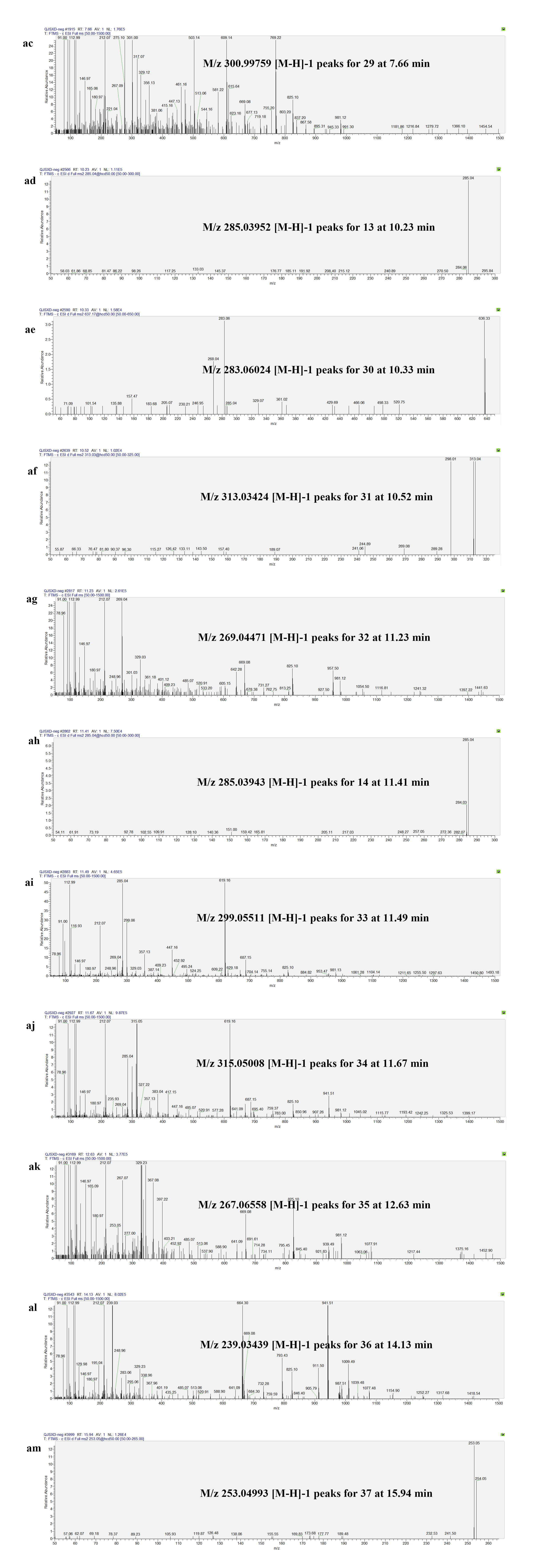

Supplement: Supplementary file 9 — Supplementary Material 9. [file 41065_2025_620_MOESM9_ESM.tif]
